# Mechanistic Insights into Drying and Film Evolution of PVA–Bentonite Coatings: The Role of Solids Content and Coating Composition Optimization

**DOI:** 10.3390/polym18162025

**Published:** 2026-08-21

**Authors:** Sarojini Verma, George D. Verros, Raj Kumar Arya

**Affiliations:** 1 Department of Chemical Engineering, Dr. B.R. Ambedkar National Institute of Technology, Jalandhar 144011, Punjab, India; sarojiniv.ch.22@nitj.ac.in; 2 Laboratory of Polymer and Dyes Chemistry and Technology, Department of Chemistry, Aristotle University of Thessaloniki (Auth), 54124 Thessaloniki, Greece; gdverros@gmail.com

**Keywords:** drying kinetics, film formation, PVA–bentonite coatings, solids content optimization, solvent diffusion, coating composition design, bentonite clay, natural filler

## Abstract

Poly(vinyl alcohol) (PVA)–bentonite composite coatings combine a hydrophilic polymer with a naturally abundant clay mineral, offering potential advantages for modifying the physicochemical and film-forming characteristics of polymer–clay coatings. However, the combined influence of PVA–bentonite composition and total solids content on drying behavior and film evolution remains insufficiently explored. This study investigates the particle size, X-ray diffraction (XRD), Fourier-transform infrared spectroscopy (FTIR), surface morphology, rheology, thixotropy, zeta potential, drying behavior, solvent transport, and film evolution of PVA–bentonite coatings prepared at total solids contents of 5 and 10 wt.% with different PVA to bentonite ratios. The drying profiles exhibited an initial relatively rapid solvent-removal stage followed by a slower stage associated with progressively restricted solvent transport during film consolidation. A lower total solids content (5 wt.%) generally accelerated drying but was associated with greater microcracking, whereas a higher total solids content (10 wt.%) produced more consolidated and comparatively uniform films with reduced solvent mobility. The combined physicochemical, rheological, drying, and morphological results demonstrate that both PVA–bentonite composition and total solids content substantially influence the structural organization and drying behavior of the coatings. Pure PVA formed a relatively uniform film but exhibited prolonged drying, while pure bentonite required the longest drying time (1083 min). Among the investigated formulations, the 50:50 PVA–bentonite coating demonstrated the shortest drying time, reaching equilibrium in approximately 480 min, while also exhibiting comparatively good film uniformity. During drying, its thickness decreased from approximately 1745 to 440 µm, corresponding to a reduction of about 1305 µm. Overall, under the investigated laboratory conditions, the 50:50 PVA–bentonite formulation provided the most favorable balance of drying behavior, film formation, and rheological characteristics among the compositions studied. These findings provide composition–structure–drying relationships that can guide further development of PVA–bentonite coating systems. At the same time, additional evaluation of mechanical, adhesion, barrier, durability, and economic performance is required to establish broader practical applicability.

## 1. Introduction

Polymeric coatings are very important in many industries, including automotive, aerospace, packaging, and electronics. They protect, improve the look of surfaces, and add barrier functionality [1]. The physical and chemical processes that occur during drying significantly impact coating performance. According to Bryntesen et al. [2], this crucial stage involves controlling the evaporation of the solvent, organizing the polymer chains, diffusion inside the material, and the consolidation of interfaces. The drying time of polymeric films is crucial and has a major impact on the final shape, stickiness, mechanical strength, and longevity of the films. Despite extensive study of composite coatings that integrate inorganic and polymeric phases, Johansson and Clegg [3] and Sapalidis et al. [4] both pointed out that the intricate relationship between composition, solids loading, and solvent transport methods remains unknown.

The use of waterborne polymeric coatings is on the rise as an eco-friendly alternative to solvent-based systems due to their lower health risks, simpler processing, and reduced emissions of volatile organic compounds (VOCs), as per Verma et al. [5]. The exceptional capabilities of poly(vinyl alcohol) (PVA), which imparts film-forming properties, mechanical flexibility, transparency, and chemical resistance, have garnered a lot of attention [6]. Due to strong hydrogen bonding with water molecules, pure PVA films exhibit slow drying rates and insufficient dimensional stability. This interaction leads to increased drying time and reduced solvent diffusion [7]. Researchers are investigating hybrid PVA–inorganic systems that include fillers like bentonite, kaolin, or silica to improve drying behavior, mechanical properties, and cost-effectiveness [8,9]. This also addresses the elevated expenses associated with pure polymer formulations.

Bentonite is naturally found and is a type of smectite clay that possesses a layered silicate structure, making it a good filler for polymeric coatings. Its usefulness stems from the intercalated nanostructures generated inside the polymer matrix, which impart a high specific surface area and ion-exchange properties [10]. As a result of strong interactions between the polymer and the clay, incorporating bentonite into the coating consequently alters its rheological properties and enhances its mechanical and barrier features [11]. Using hydrophobically modified PVA–bentonite films, Bemada and Bruneau [12] found that barrier characteristics and permeability were significantly enhanced. Issa et al. [13] also discovered that PVA–bentonite nanocomposites had reduced gas permeability and a higher modulus. Based on their review of polymer–clay nanocomposites, Lee and Choi [14] concluded that moderate bentonite loading increases stiffness, decreases water permeability, and maintains flexibility. Taken as a whole, the results suggest that PVA–bentonite coatings could be a high-performance and environmentally friendly substitute for conventional polymeric systems.

Despite these advancements, many previous studies on PVA–bentonite coatings have focused on their surface mechanical or barrier properties. In contrast, very few studies have focused on their drying kinetics or film-evolution behavior. The drying process of multicomponent systems becomes more complex due to the transport of solvents through a constantly changing matrix [15,16,17]. This matrix also interacts with fillers and changes as the polymer relaxes and concentration gradients move together. Classical drying theory recognizes two primary phases: the constant-rate phase, where surface evaporation occurs when the film is saturated with solvent, and the falling-rate phase, where the limiting factor is solvent diffusion through the semi-solid network [1,6,18,19,20,21,22]. Composite coatings have an earlier transition due to micro-capillary effects and winding diffusion pathways caused by distributed bentonite layers [7,8]. Bentonite slurries show faster solvent removal in the beginning, but more diffusion resistance later due to particle aggregation and microstructural rearrangement [8]. Adding solid particles changes the capillary dynamics, which could stabilize the liquid layer and prevent breaking [7].

The drying kinetics of coatings are significantly influenced by the concentration of particulates in the coatings. Studies carried out by Arjmandi et al. [23], Cairncross et al. [24], and Jain et al. [25] reported that elevated solids content reduced film shrinkage, defects, and solvent diffusion, while, on the other hand, it enhanced viscosity as a consequence of a higher proportion of non-volatile substances in the matrix. By contrast, a decrease in particulate content accelerates the drying process; however, it can also result in structural damage or porousness in films. Research indicates that the solids loading and polymer–filler interactions in composite systems are responsible for the mechanisms of solvent transport and the evolution of film density [9,10,13]. Depending on the solids content, the drying profiles of clay-based polymer coatings vary, as per Nechita [9]. Coatings with lesser solids loadings exhibited greater surface homogeneity, while coatings with higher solids loadings exhibited less porosity. Regulating the solids content and composition will lead to achieving balanced coating qualities.

Recent developments in experimental characterization and modeling allow for a deeper investigation of the mechanisms. The substantial impact of composition on the rate of film densification and solvent activity is indicative of the influence of the diffusion–evaporation model explored by Favre et al. [26] and Huisman et al. [27]. Shalygina et al. [28] demonstrated through their study that the filler’s morphology and the particle aspect ratio affect the mechanical consolidation and diffusion resistance of hybrid coatings. These factors interact to determine the residual solvent content, film thickness evolution, and coating drying kinetics, all of which have an impact on performance.

The purpose of this study is to elucidate the mechanism governing the drying kinetics and film development of PVA–bentonite coatings with 5% and 10% total solids contents. The coating compositions varied in the ratios of PVA to bentonite used for making the solutions (pure PVA, pure bentonite, and mixtures of PVA and bentonite at ratios of 75/25, 50/50, and 25/75). The amounts of PVA and bentonite, residual solvent, and coating thickness were measured in order to comprehend the drying transitions and identify the crucial steps in solvent removal. Solvent transport, structural consolidation, and the economic implications in polymer–clay coatings are thoroughly examined in this work.

## 2. Materials and Methods

Sodium bentonite (i.e., montmorillonite) powder and commercial-grade polyvinyl alcohol (PVA) were used in this study. Aaradhya Pulp & Minerals, Kanpur, India, supplied the bentonite powder, which was imported from Turkey. The bentonite used in this study was sodium bentonite, with a high swelling value (26 mL/2 g), 325 Mesh size, pH value of 9.35, Humidity of 7–10%, and plate water absorption of 550%. It has a molecular weight of 180.06 g/mol and a density of 0.593 g/cm^3^. The polyvinyl alcohol, with a molecular weight of 86.09 g/mol, density of 1.19 g/cm^3^, and degree of hydrolysis of 86–89%, was supplied by Lobachemie, Mumbai, India. All chemicals were used without any pre-treatment.

### 2.1. Preparation of PVA–Bentonite Coatings

The poly(vinyl alcohol) and bentonite mixtures were prepared in order to obtain the following compositions: 100% PVA, 75% PVA–25% bentonite, 50% PVA–50% bentonite, 25% PVA–75% bentonite, and 100% bentonite. These weighed amounts were mixed using a food processor (DYNAMIX, a Heavy Duty Mixer Grinder, Mittal Electronics, New Delhi, India, Maximum rpm 22,000) at medium speed for 10 min, followed by manual mixing using a food processor at 700 rpm for 10 min, in order to achieve uniform mixing of the clay and the polymer. These mixtures were stored in an air-tight glass jar for further use.

In order to prepare the suspensions of these mixtures, around 5 g of the mixture was weighed and poured into 95 g of deionized water in leak-proof glass bottles, to obtain 5% solid and 95% deionized water. These sealed bottles were mechanically shaken in an orbital shaker (REMI CIS-18 Plus, Remi, New Delhi, India) at 200 rpm at 40 °C for 8 days in order to obtain uniform suspensions. The mixtures were ultrasonically treated for one hour to eliminate any air bubbles and homogenized using a Cole Parmer UC 200 ultrasonic bath sonicator (Cole-Parmer, Shanghai, China).

In order to prepare the coatings, the previously prepared solution was poured into a cylindrical sample holder with a diameter of 19 mm and depth of 5 mm. A micropipette was used to pour the solution into the solution holder. The sample holders were made up of mild steel and were available in various depths, i.e., 0.5 mm, 1 mm, 2 mm, etc. Before pouring the solution into the sample holder, the sample holder was placed on a computerized analytical balance (Radwag, 82/220 X2 Plus, Radom, Poland) with an accuracy of ±0.0001 g in continuous transmission mode. The solution was poured into the holder after recording the mass of the empty sample holder to observe the drying pattern of the solution (i.e., polymer suspension) at intervals of one minute until a stable weight was achieved, typically 10–14 h due to the slow evaporation of water from the suspension. During the course of drying, water evaporated into the surrounding air without any air flow at room temperature (25 ± 2 °C). The coating thickness, residual solvent, and concentrations were calculated using volumetric analysis. Similar calculations have been reported by Pathania et al. [29].

The calculations for taking aliquots from the stock solutions followed the calculations described in the example below. Suppose the mass of the coating suspension on the substrate (sample holder with a diameter 19 mm and depth of 2 mm) was 2 g for the suspension with 5% solids (75%PVA–25% bentonite mixture) and 95% solvent (i.e., water in the present case). The total mass of solids at any given instant in the aliquot is calculated as follows:

(1) $$\left[\begin{array}{c} Total\;mass\;of\;solids\;\\ in\;the\;coating\;suspension \end{array}\right]=2\;g\times \frac{5}{100}=0.1\;g$$

(2) $$\left[\begin{array}{c} Mass\;of\;PVA\;\\ in\;the\;coating \end{array}\right]=\left[\begin{array}{c} Total\;mass\;of\;solids\\ in\;the\;coating\;suspension \end{array}\right]\times \frac{75}{100}=0.1\;g\times \frac{75}{100}=0.075\;g$$

(3) $$\left[\begin{array}{c} Mass\;of\;bentonite\;\\ in\;the\;coating \end{array}\right]=\left[\begin{array}{c} Total\;mass\;of\;solids\\ in\;the\;coating\;suspension \end{array}\right]\times \frac{25}{100}=0.1\;g\times \frac{25}{100}=0.025\;g$$

(4) $$\left[\begin{array}{c} Mass\;of\;water\;\\ in\;the\;coating \end{array}\right]=2\;g\times \frac{95}{100}=1.9\;g$$

(5) $$\left[\begin{array}{c} Volume\;of\;PVA\;\\ in\;the\;coating \end{array}\right]=\frac{\mathrm{mass}\;\mathrm{of}\;\mathrm{PVA}\;\mathrm{in}\;\mathrm{the}\;\mathrm{coating}}{\mathrm{Density}\;\mathrm{of}\;\mathrm{PVA}}=\frac{0.075\;g}{1.19\;\frac{g}{cm^{3}}}=0.0630\;cm^{3}$$

(6) $$\left[\begin{array}{c} Volume\;of\;bentonite\;\\ in\;the\;coating \end{array}\right]=\frac{\mathrm{mass}\;\mathrm{of}\;\mathrm{bentonite}\;\mathrm{in}\;\mathrm{the}\;\mathrm{coating}}{\mathrm{Density}\;\mathrm{of}\;\mathrm{bentonite}}=\frac{0.025\;g}{0.593\;\frac{g}{cm^{3}}}=0.0422\;cm^{3}$$

(7) $$\left[\begin{array}{c} Volume\;of\;water\;\\ in\;the\;coating \end{array}\right]=\frac{\mathrm{mass}\;\mathrm{of}\;\mathrm{water}\;\mathrm{in}\;\mathrm{the}\;\mathrm{coating}}{\mathrm{Density}\;\mathrm{of}\;\mathrm{water}}=\frac{1.9\;g}{0.9970\;\frac{g}{cm^{3}}}=1.9057\;cm^{3}$$

(8) $$\left[\mathrm{Volume}\;\mathrm{of}\;\mathrm{the}\;\mathrm{coating}\right]=1.9057\;cm^{3}+0.0422\;cm^{3}+0.0630\;cm^{3}=2.0109\;cm^{3}$$

(9) $$\text{Cross-sectional}\;\mathrm{area}\;\mathrm{of}\;\mathrm{coating}=\frac{\pi }{4}{\left(\mathrm{diameter}\right)}^{2}=\frac{22}{7\times 4}\times {\left(1.9\;cm\right)}^{2}=2.8364\;cm^{2}$$

(10) $$\left[\begin{array}{c} Instantaneous\;coating\;\\ thickness \end{array}\right]=\frac{\mathrm{coating}\;\mathrm{volume}}{\mathrm{cross}\;\sec\mathrm{tional}\;\mathrm{area}}=\frac{2.0109\;cm^{3}}{2.8364\;cm^{2}}=0.709\;cm=0.709\;cm\times \frac{10000\;\mu m}{1\;cm}=7090\;\mu m$$

(11) $$\left[\begin{array}{c} Instantaneous\;concentration\\ of\;PVA \end{array}\right]=\frac{\mathrm{mass}\;\mathrm{of}\;\mathrm{PVA}}{\mathrm{volume}\;\mathrm{of}\;\mathrm{coating}}=\frac{0.075\;g}{0.063\;cm^{3}}=0.0373\;\frac{g}{cm^{3}}$$

(12) $$\left[\begin{array}{c} Instantaneous\;concentration\\ of\;bentonite \end{array}\right]=\frac{\mathrm{mass}\;\mathrm{of}\;\mathrm{bentonite}}{\mathrm{volume}\;\mathrm{of}\;\mathrm{coating}}=\frac{0.025\;g}{2.0109\;cm^{3}}=0.0124\;\frac{g}{cm^{3}}$$

(13) $$\left[\begin{array}{c} Instantaneous\;concentration\\ of\;water \end{array}\right]=\frac{\mathrm{mass}\;\mathrm{of}\;\mathrm{water}}{\mathrm{volume}\;\mathrm{of}\;\mathrm{coating}}=\frac{1.9\;g}{2.0109\;cm^{3}}=0.9449\;\frac{g}{cm^{3}}$$

(14) $$\left[\begin{array}{c} Instantaneous\\ \%\;residual\;solvent \end{array}\right]=\frac{\mathrm{instantaneous}\;\mathrm{mass}\;\mathrm{of}\;\mathrm{solvent}}{\mathrm{initial}\;\mathrm{mass}\;\mathrm{of}\;\mathrm{solvent}}=\frac{1.9\;g}{1.9\;g}\times 100=100\%$$

Suppose the coating mass after 1 min is 1.9 g. In order to perform the above calculations, the mass of PVA and the mass of bentonite will remain unchanged due to their non-volatile nature; only the mass of water will change due to evaporation.

(15) $$\left[\begin{array}{c} Mass\;of\;water\;\\ in\;the\;coating \end{array}\right]=\underset{\begin{array}{c} current\;mass\\ of\;coating \end{array}}{\underbrace{1.9\;g}}-\underset{\mathrm{mass}\;\mathrm{of}\;\mathrm{PVA}}{\underbrace{0.075\;g}}-\underset{\mathrm{mass}\;\mathrm{of}\;\mathrm{bentonite}}{\underbrace{0.025\;g}}=1.8\;g$$

Now we can repeat the calculations from Equations (5)–(14) in order to obtain the instantaneous concentrations, thickness, and residual solvent.

The rheological properties of the polymer and coating formulations were analyzed using a modular compact rheometer with a parallel plate geometry of 25 mm at a strain rate of 1 to 100 s^−1^ [30,31,32]. The flow behavior was observed at 25 °C for any changes. Hysteresis curves were recorded at incremental strain rates of 5 s^−1^ to 100 s^−1^, held for 100 s^−1^, and decreased from 100 s^−1^ to 5 s^−1^ at 25 °C, with a pre-shear step of 5 s^−1^. This method was adopted to ensure that the microstructure always started the same way and to erase any previous shear history. After that, steady-shear tests were performed at different shear rates. We used the parallel-plate shape because the solutions used to make the film were thick, which meant that the shear conditions were the same over the entire sample. We analyzed the rheological data to determine if Newtonian or shear-thinning behavior occurred. This would alter the application of the coating, the mixing of the solution, and the fabrication of the film [33,34].

### 2.2. Material Characterizations

Elemental analysis and surface morphology analysis were performed using a Field Emission Scanning Electron Microscope with EDS (FESEM SIGMA 500VP, ZEISS, Oberkochen, Germany). Phase analysis of the prepared films, pure PVA, and bentonite was performed using an X-ray diffractometer (EMPYREAN, PANalytical, Malvern Panalytical Ltd., Malvern, UK). Thixotropy and rheology analyses were performed using a rheometer (MCR92, Anton Paar, Ostfildern, Germany). Functional group analysis of the pure powders and films was performed using FTIR (PerkinElmer Spectrum 2, Shelton, CT, USA). Free swelling analysis was conducted using a standard process (ASTM D5890) [35], which involved checking the swelling volume of 2 g of dry bentonite powder in 100 mL of deionized water in a measuring cylinder. Particle size and zeta potential analyses were performed using dynamic light scattering (90 PLUS particle size analyzer and zeta potential analyzer, Brookhaven, Holtsville, NY, USA).

## 3. Results and Discussion

The coatings with varying PVA to bentonite ratios were synthesized with 5% and 10% total solids contents to observe the effect on the solvent evaporation rate, solvent retention degree, and coating consolidation process. Due to its interaction with bentonite, which acts as a diffusion barrier, the polymeric PVA binder influences the drying efficiency and overall film shape. Mass loss, solvent percentage, concentration, and thickness changes over time were quantitatively measured to determine the kinetic regimes and transition points related to solvent diffusion and film development.

The formulations were prepared at total solids contents of 5 and 10 wt.%, with PVA to bentonite mass ratios of 100:0, 75:25, 50:50, 25:75, and 0:100. These composition ratios were selected to systematically represent the full range from pure PVA to pure bentonite while limiting the number of formulations required for comparative evaluation. The total solids content was restricted to a maximum of 10 wt.% to maintain adequate flowability. This percentage of solids also enables uniform casting and spreading over the substrate surface, and based on a previous study [5,36], increasing the total solids content beyond 10 wt.% results in substantially higher viscosity. Therefore, 5 and 10 wt.% were selected to investigate the effect of solids loading while maintaining reproducibility.

### 3.1. Particle Size Analysis of Films

**Figure 1:**
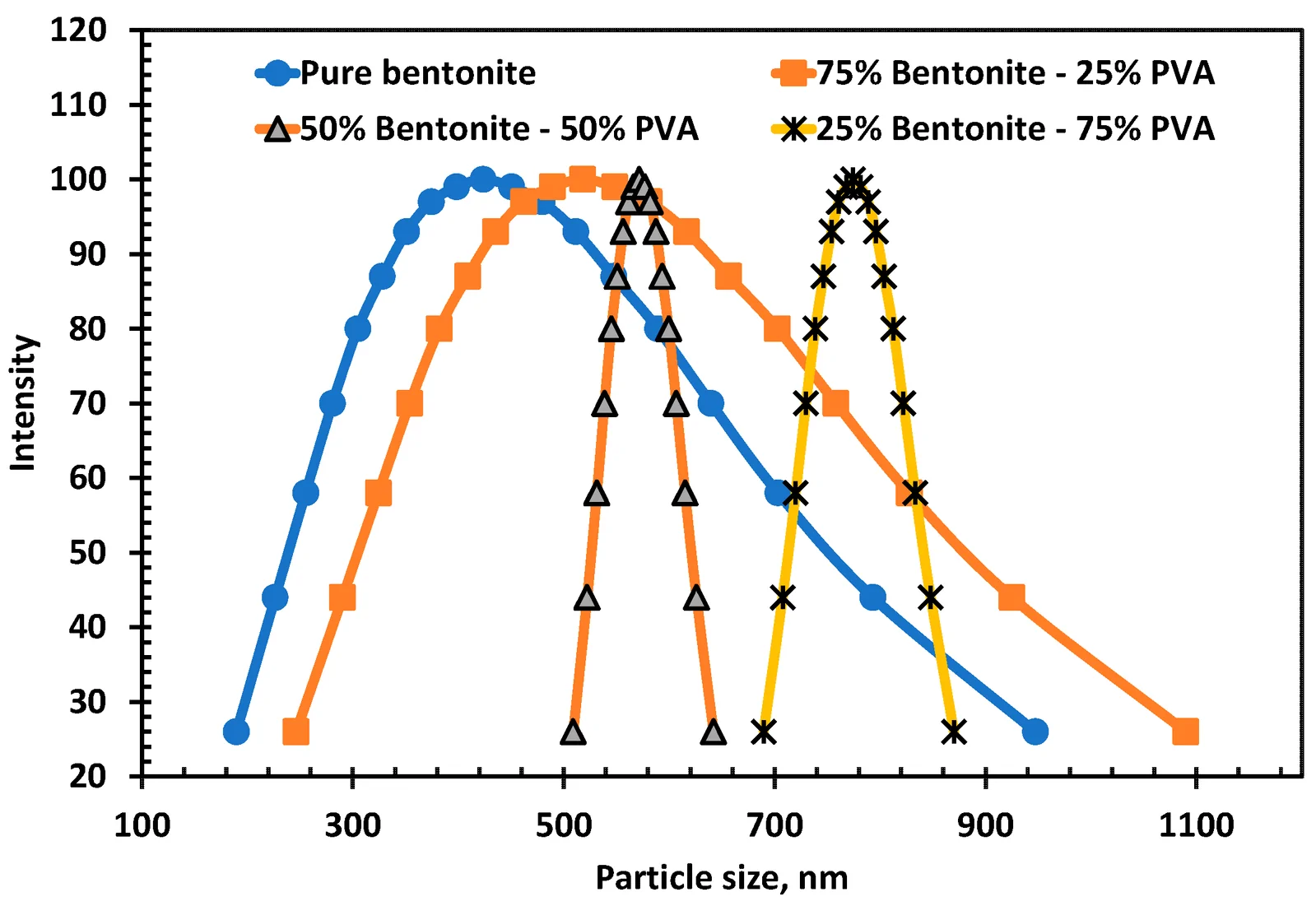
DLS analysis of pure bentonite and various bentonite–PVA blend dispersions.

Figure 1 shows the particle size analysis of various bentonite–PVA dispersions. Variations in the hydrodynamic size and distribution were observed with changes in PVA content, demonstrating that the composition of the PVA–bentonite system influences the apparent particle size characteristics of the aqueous dispersion. The pure bentonite suspension had a Polydispersity Index (PDI) of 0.27 and an average particle size of 477.7 nm. This indicates a relatively narrow particle size distribution, which is consistent with the characteristics of aqueous dispersions of layered silicate clays. In these types of dispersions, individual clay platelets may co-exist with partially associated tactoids. Electrostatic interactions involving the charged basal surfaces and platelet edges can influence the association and dispersion of clay particles in aqueous media. Therefore, the observed hydrodynamic size may reflect contributions from both individual or partially dispersed platelets and tactoid structures. However, particle size analysis alone cannot directly distinguish between these structural arrangements.

When PVA was added, the PDI slowly decreased, and the effective particle size increased for the 25% PVA–75% bentonite formulation. The PDI of the particles was 0.227, with an effective particle diameter of 573.9 nm. The PVA chains attach to the clay surface due to hydrogen bonds formation between the hydroxyl groups in the PVA and the silanol and aluminol groups in the clay. The polymer adsorption shields the charge on the surface of the clay to some extent, lowers the interactions between clay particles, and yields a more uniform dispersion in comparison to pure bentonite dispersions.

In the 50:50 formulation, the average hydrodynamic particle size was 573.1 nm, which was comparable to other bentonite-containing formulations. The PDI, on the other hand, decreased to 0.005, indicating a highly narrow particle size distribution under the measurement conditions. Due to the small size range, the polymer and clay interact strongly and evenly, which keeps the particles from aggregating and greatly stabilizes the suspension. However, these parameters alone cannot establish the extent of the interactions.

In the polymer-rich regime (75%PVA–25% bentonite), the average particle size dramatically increased to 776.6 nm, although the PDI stayed extremely low at 0.005. The thicker PVA layers adhering to the clay increase the hydrodynamic size. The polymer may also bind clay particles that are extremely close to one another. The polymer–clay compounds enlarge without changing their appearance. Despite their larger size, the low PDI indicates that the particles are adhering to one another in a controlled manner.

These findings demonstrate that PVA effectively adheres to the bentonite surface, gradually enlarging and dispersing the particles. PVA mostly modifies the surface and strengthens the steric structure at low to medium concentrations. With an increase in concentration, the polymer saturates the interface and its form is modified. This enlarges the hydrodynamic diameter. The surface orientation of PVA on the bentonite platelets is best described as an adsorbed and hydrogen-bonded layer featuring loop-tail shapes that reach into the water phase [37,38,39,40,41]. This configuration keeps the mixtures of clay and polymer stable and homogeneous.

### 3.2. Free Swelling Analysis of Bentonite–PVA Blends

The free swelling volume (FSV) test was used to investigate how much water the pure PVA, pure bentonite, and bentonite–PVA mixtures could hold and increase in volume. ASTM D5890 was used to determine the free swelling index, which characterizes the extent to which clay particles expand upon hydration due to interlayer water uptake. The free swelling index was determined using powdered samples to characterize the intrinsic swelling behavior of bentonite upon hydration under unconstrained conditions. The use of the powdered form minimized the influence of film structure and substrate constraints, allowing the hydration-induced expansion of the clay to be evaluated independently. Bentonite exhibits appreciable swelling primarily because of water uptake within and between its layered structure.

By contrast, PVA is hydrophilic and can absorb water through its hydroxyl groups, but it does not exhibit the same clay-like interlayer swelling mechanism. Accordingly, the ASTM D5890 free swelling index is applicable to characterize the swelling behavior of bentonite and does not directly quantify the volumetric swelling of PVA. The swelling characteristics obtained from this test therefore provide information on the hydration response of the clay component, which can be considered alongside the rheological and film-formation results when evaluating the behavior of the PVA–bentonite coatings.

Briefly, 2.0 g of oven-dried sample was added to a 100 mL graduated cylinder containing distilled water at room temperature (25 ± 2 °C) and was left untouched for 24 h in order to avoid external mixing or forceful dispersion. After equilibration, the total swollen volume was measured directly using the graduated cylinder. The free swelling index was then expressed as the swollen volume per unit mass of dry sample (mL g^−1^).

**Figure 2:**
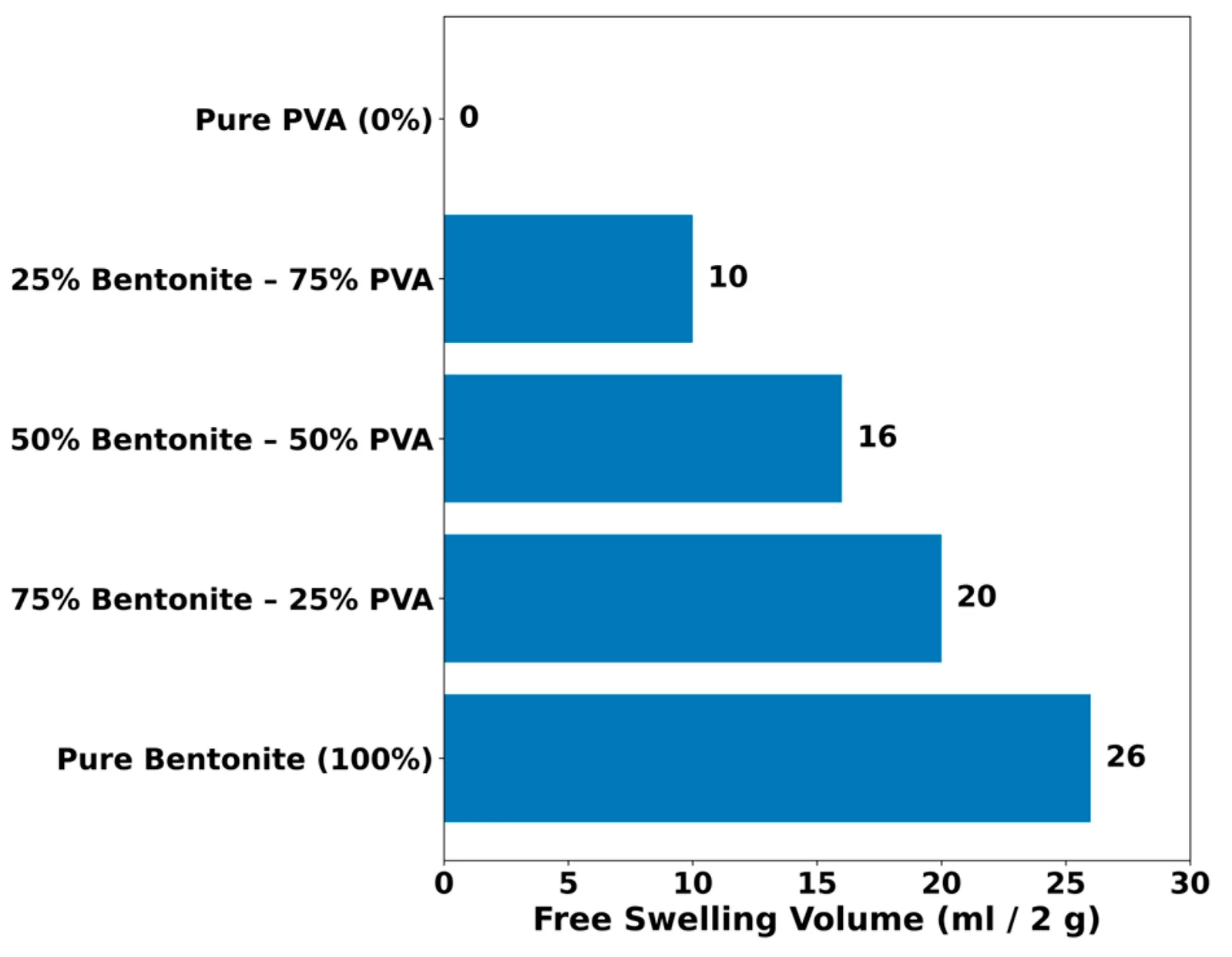
Free swelling volume for various bentonite–PVA blends.

The free swelling values for several blends of bentonite and PVA are displayed in Figure 2. The free swelling volume (FSV) tests showed that the addition of bentonite modified the poly(vinyl alcohol) (PVA). According to the data from the study, pure bentonite had an extremely high free swelling volume (FSV) of 26 mL/2 g due to the montmorillonite platelets soaking up water and expanding, whereas PVA displayed no such swelling characteristics.

These results demonstrate how PVA inhibits the swelling of bentonite. When the combination was 75% PVA and 25% bentonite, the FSV significantly decreased to 10 mL/2 g. This represents a reduction of over 62% compared to pure bentonite. This large drop indicates that the polymer chains adhere to the clay surface quite firmly at high PVA concentrations. As a result, water finds it difficult to enter the interlayer galleries.

The free swelling behavior increased with increasing bentonite content. The 50% bentonite–50% PVA formulation exhibited a free swelling volume of 16 mL per 2 g of dry sample, whereas the 75% bentonite–25% PVA formulation showed a higher value of 20 mL per 2 g. This trend indicates that the relative proportion of bentonite strongly influences the swelling response of the composite dispersions. The higher swelling observed at greater clay loading is consistent with the greater contribution of the hydrated clay phase to the overall swelling response.

By comparison, the incorporation of PVA reduced the measured free swelling relative to the clay-rich formulation. This behavior may be associated with interactions between PVA chains and the bentonite surface, which can modify the hydration and association behavior of the clay particles. However, the free swelling test alone cannot establish the specific nature of these interactions, such as hydrogen bonding, the location of polymer adsorption, or changes in interlayer water accessibility. The observed reduction in swelling should therefore be considered an indication of a modified clay hydration response rather than direct evidence of a specific polymer–clay interaction mechanism.

Overall, the free swelling results demonstrate that increasing the bentonite fraction increases the swelling capacity of the PVA–bentonite system under the conditions of the ASTM D5890 test. These results provide useful information on the hydration behavior of the clay component. They can be considered alongside the rheological and film-formation results when interpreting the composition-dependent behavior of the coatings.

### 3.3. FTIR Analysis of Bentonite–PVA Films

**Figure 3:**
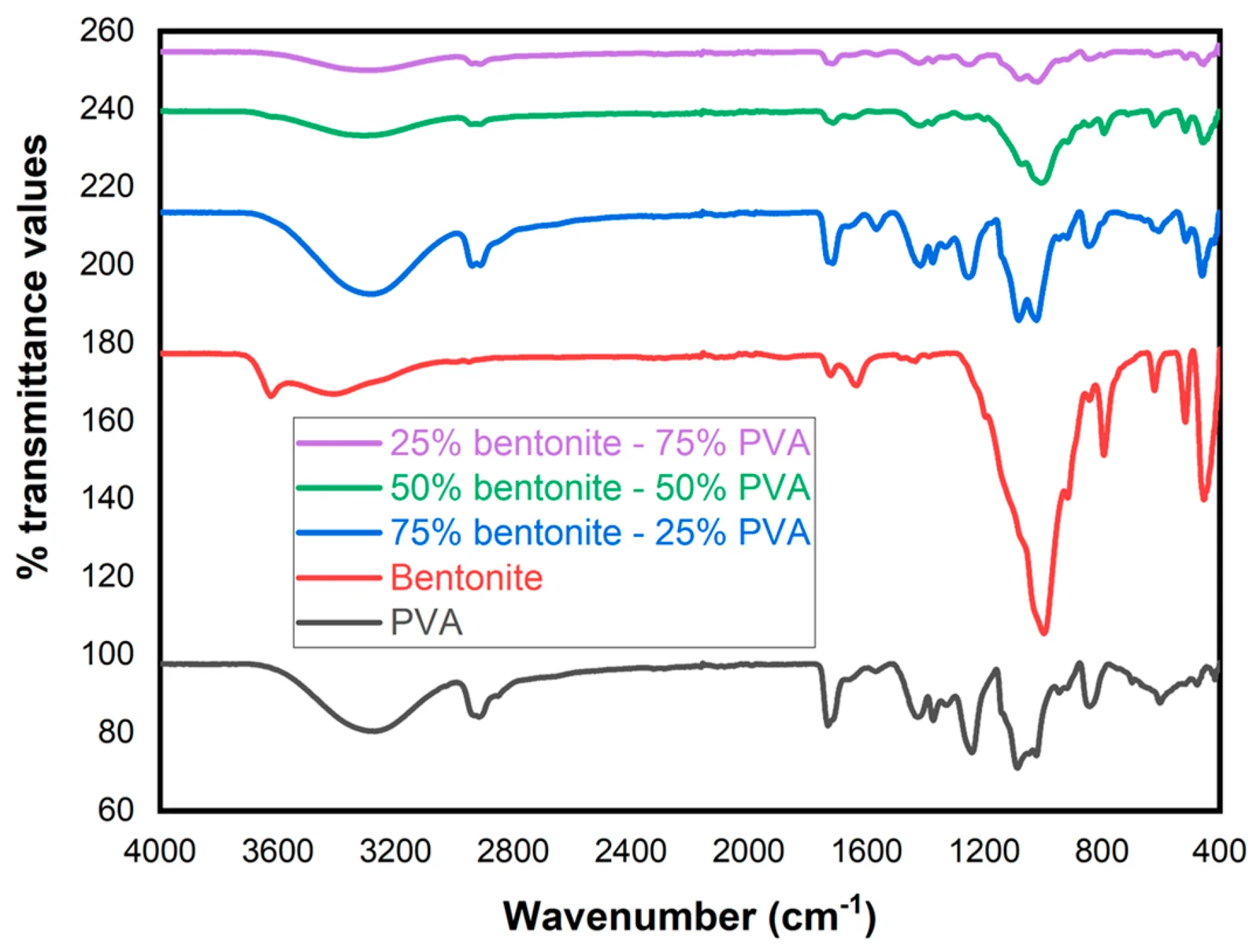
Cumulative FTIR spectra of bentonite, PVA, and their blend films.

Figure 3 shows the FTIR spectra of the PVA, bentonite, and various bentonite–PVA blend coatings. The spectrum of pure PVA exhibited a broad band at 3272 cm^−1^, which corresponds to the O–H stretching vibration of the abundant hydroxyl groups of PVA reported in earlier studies [42,43]. The absorption band at 2917 cm^−1^ was assigned to C–H stretching vibrations of the PVA backbone. A relatively weak band observed at 1731 cm^−1^ can be attributed to the C=O stretching vibrations associated with residual acetate groups, consistent with the degree of hydrolysis of the PVA used in this study. The band at 1374 cm^−1^ was assigned to C–H bending, the band at 1242 cm^−1^ indicated the C–O stretching of acetate groups, and the band at 1087 cm^−1^ was associated with the C–O stretching of secondary alcohols. The lower wavenumber bands at 841 cm^−1^ and 603 cm^−1^ were associated with C–C stretching and bends that show skeletal and deformation vibrations of the PVA structure. Overall, the FTIR spectrum confirms the characteristic functional groups of PVA, particularly its hydroxyl-rich structure.

The FTIR spectrum of pure bentonite exhibited a characteristic absorption band at 3624 cm^−1^, assigned to the structural O–H stretching associated with hydroxyl groups in the octahedral sheets. The band at 1634 cm^−1^ was attributed to the bending vibration of adsorbed/interlayer water. Additional absorption features at 1435 and 1722 cm^−1^ may be associated with minor carbonate-containing or other accessory species present in the natural clay. A strong absorption band at 997 cm^−1^ was assigned to the Si–O stretching vibrations of the silicate framework. Bands observed at 793, 622, 517, and 455 cm^−1^ were associated with the characteristic Si–O and Al–O–Si/Si–O–Si deformation vibrations of the aluminosilicate structure. These spectral features are consistent with the characteristic functional groups of bentonites.

The FTIR spectra of the PVA–bentonite composite coatings contained characteristic absorption features originating from both PVA and bentonite, with their relative intensities varying according to composition. For the 75% bentonite–25% PVA formulation, a broad O–H stretching band was observed at approximately 3280 cm^−1^, together with a C–H stretching band at approximately 2940 cm^−1^ and a carbonyl-related band at approximately 1716 cm^−1^. The strong absorption features within 1083–1023 cm^−1^ contained contributions from the silicate structure of bentonite, while characteristic aluminosilicate deformation bands remained observable at lower wavenumbers. The simultaneous presence of PVA- and bentonite-associated bands confirms the presence of both components in the composite coating.

For the 50% bentonite–50% PVA formulation, a broad O–H stretching band was observed at approximately 3305 cm^−1^. Characteristic PVA-related absorption bands were also observed at approximately 2943 cm^−1^ (C–H stretching), 1420 cm^−1^ (CH_2_ bending), and 1268 cm^−1^ (C–O-related vibrations), together with the characteristic silicate absorption at approximately 1008 cm^−1^ and aluminosilicate deformation bands at lower wavenumbers. The coexistence of these characteristic spectral features indicates that both PVA and the bentonite silicate framework remain present in the composite.

In the 25% bentonite–75% PVA formulation, PVA-associated spectral features became more prominent, consistent with its higher proportion in the formulation. A broad O–H stretching band occurred at approximately 3298 cm^−1^, while the band at approximately 1719 cm^−1^ was assigned to carbonyl-containing residual acetate groups. The 1019–1080 cm^−1^ region contained overlapping contributions from the C–O stretching of PVA and the Si–O stretching of bentonite, making the individual contributions difficult to resolve unequivocally in the composite spectrum.

Overall, the FTIR analysis confirms the characteristic functional groups of both PVA and bentonite in the composite coatings. Changes in the position, width, and relative intensity of the O–H stretching region with composition are consistent with changes in the local hydrogen-bonding environment associated with the hydroxyl-containing PVA and bentonite phases. However, these spectral changes alone do not provide direct evidence for the formation or strength of specific PVA–bentonite hydrogen bonds. Furthermore, no conclusions regarding nanoscale dispersion, intercalation, polymer-chain mobility, or mechanical and barrier performance can be drawn solely from the FTIR data. These structural and performance characteristics require complementary characterization.

The broad O–H stretching region associated with PVA (approximately 3310–3270 cm^−1^) and the structural –OH groups of bentonite (approximately 3620 cm^−1^) exhibited changes in band position and broadening in the composite coatings. These spectral changes are consistent with the modification of the local hydrogen-bonding environment following the incorporation of PVA and bentonite and may reflect interactions between the hydroxyl-containing groups of PVA and the surface sites of bentonite. However, the observed shifts and broadening are not considered direct proof of the formation or increased strength of specific interfacial hydrogen bonds. The persistence of the carbonyl-related band at approximately 1730–1715 cm^−1^ indicates the presence of residual acetate groups associated with the PVA used in this study. Characteristic Si–O stretching bands (approximately 1030–1000 cm^−1^) and Al–O–Si deformation bands remained observable in the composite spectra, indicating retention of the characteristic aluminosilicate framework of bentonite. Changes in band position, width, and relative intensity within the overlapping 1100–1000 cm^−1^ region may arise from changes in the local chemical environment of PVA and bentonite functional groups upon composite formation. Similar spectral changes associated with polymer–clay interactions have been reported for PVA–clay and other polymer-layered silicate systems [33,34,44].

### 3.4. Zeta Potential Analysis of Bentonite–PVA Dispersions

For the zeta potential measurements, aliquots of the original 5 and 10 wt.% PVA–bentonite formulations were diluted to a total solids content of 2 wt.% using deionized water while maintaining the original PVA to bentonite ratio of each formulation. Dilution was necessary because the undiluted 5 and 10 wt.% dispersions exhibited excessive turbidity, which limited light transmission and prevented reliable optical measurements. The observed results are consistent with interactions between PVA and bentonite at the solid–liquid interface. These interactions may involve the adsorption of PVA chains onto the basal surfaces or edge sites of the bentonite particles, as reported for similar polymer–clay systems [45,46,47]. The zeta potential of the pure bentonite dispersion was observed to be −18.77 mV. This is consistent with the dispersion characteristics commonly observed for montmorillonite-type clays in dilute aqueous solutions. The primary cause of this negative value is the persistent negative charge on the basal (facial) surfaces due to isomorphic substitution in the clay lattice. This number is also influenced by charges on the edges that vary with pH. The negatively charged faces push one another away at this solids concentration, preventing the dispersion from disintegrating.

For the 75% bentonite–25% PVA dispersion, the zeta potential shifted significantly from neutral to −3.01 mV. The observed decrease in the magnitude of the zeta potential upon PVA addition may be associated with the adsorption of PVA chains onto accessible clay surfaces, which can modify the interfacial properties of the bentonite particles. The silanol and aluminol groups on the surface of bentonite and the hydroxyl groups in PVA create hydrogen bonds that mask the negative charges on the basal faces. When charges are attached to the edges, interactions with the edge sites work against them. The electrical double-layer compression is very strong when both the face and edge surfaces are covered.

The zeta potential of the 50% bentonite–50% PVA dispersion was −8.56 mV, which means that it was in a medium-charge state. This behavior indicates that the PVA adheres more uniformly to both the face and edge surfaces of the clay platelets. PVA changes the chemistry of the surface, but some of the basal surface charge remains, which makes the zeta potential slightly negative. In this case, the dispersion’s stability depends on the residual electrostatic repulsion and the strong steric stabilization provided by the polymer layer that has been adsorbed.

The zeta potential of the 25% bentonite–75% PVA dispersion was −3.74 mV, which means a nearly neutral charge. At this high polymer concentration, there is a lot of PVA on top of the clay particles, and thick layers of polymer form around them. The PVA molecules adhere to the edges and the basal faces at the same time. Excess polymer enters the water phase, which screens the surface charges and moves the stabilization mechanism toward steric control.

The zeta potential results indicate that the presence of PVA alters the interfacial charge characteristics of the bentonite-containing dispersions. Compared to pure bentonite, the incorporation of PVA generally decreased the magnitude of the zeta potential, although this trend was not strictly monotonic across all PVA to bentonite ratios. This change is consistent with the modification of the bentonite–water interface and may be associated with PVA adsorption onto accessible clay surfaces, as reported previously for PVA–bentonite systems [48,49]. However, the zeta potential measurements alone cannot confirm the specific adsorption sites or molecular configuration of PVA on the bentonite surface.

Zeta potential graphs for various samples are given in Figures S1–S4 in the Supplementary Materials.

### 3.5. XRD Analysis of Bentonite–PVA Coatings

**Figure 4:**
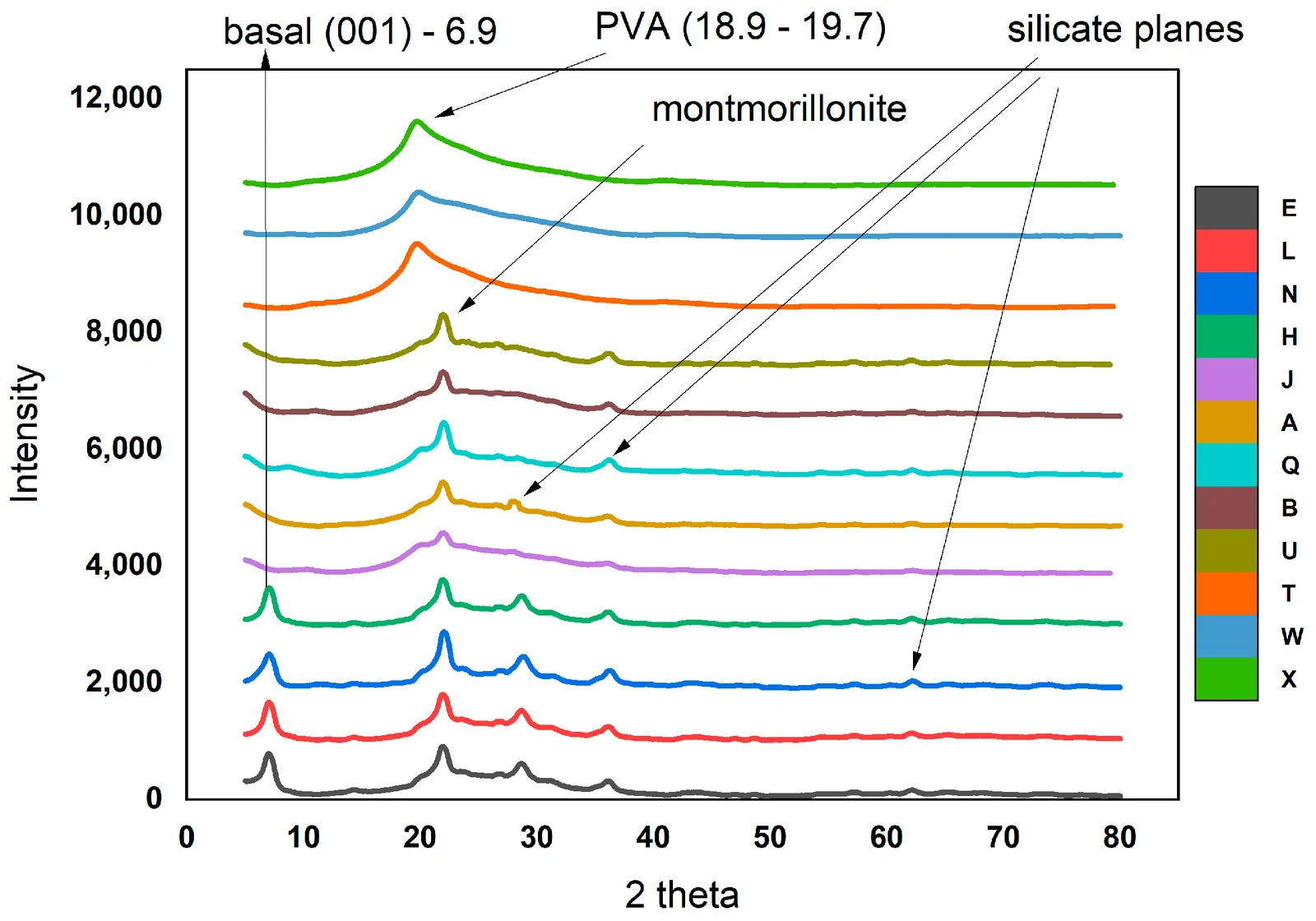
XRD diffractograms of pure PVA powder, bentonite powder, and their coatings (A: 5% coating of 50% bentonite–50% PVA, B: 5% coating of 75% bentonite–25% PVA, E: pure bentonite powder, H: 5% coating of 25% bentonite–75% PVA, J: 10% coating of 25% bentonite–75% PVA, L: 5% coating of pure bentonite, N: 10% coating of pure bentonite, Q: 10% coating of 50% bentonite–50% PVA, T: 5% coating of pure PVA, W: 10% coating of pure PVA, X: 10% coating of 75% bentonite–25% PVA).

X-ray diffraction (XRD) was used to investigate composition- and solids content-dependent structural changes in pure PVA, pure bentonite, and PVA–bentonite composite coatings prepared at 5 and 10 wt.% total solids (Figure 4). The diffraction patterns provide information on the crystalline and layered structural characteristics of the constituent phases and their changes with composition. These structural variations may be related to differences in film formation and drying behavior; however, the XRD results alone cannot directly establish polymer–clay compatibility, nanoscale dispersion, intercalation, or specific solvent-transport pathways.

The XRD pattern of pure bentonite exhibited the pronounced reflections characteristic of a montmorillonite-rich layered aluminosilicate structure. The low-angle basal (001) reflection appeared at approximately 2θ ≈ 7.3°, while additional reflections were observed at approximately 21–22°, 27–30°, 36–39°, and 62–72°. These reflections are associated with the crystalline and layered mineral phases present in the bentonite. Increasing the total solids content from 5 to 10 wt.% resulted in sharper and more pronounced diffraction features, together with a reduction in the relative background contribution. This change is consistent with the increased contribution of the solid phase to the diffraction pattern. It may also reflect differences in the packing or structural organization of the clay particles. However, the XRD data do not by themselves establish changes in tactoid packing or microstructural robustness.

Pure PVA exhibited the characteristic semi-crystalline reflection at approximately 2θ ≈ 18.5–19.7°. The broad nature of this reflection, particularly at 5 wt.% solids, indicates relatively limited crystalline ordering within the predominantly amorphous PVA matrix. At 10 wt.% solids, the PVA-associated reflection became more pronounced, suggesting an increase in the detectable degree of structural ordering with increasing solids content. The changes in PVA crystallinity may influence the physical structure of the dried coating; however, a direct relationship between the XRD features and solvent mobility or drying rate cannot be established from XRD alone.

The diffraction patterns of the PVA–bentonite composite coatings reflected contributions from both constituent phases, with the relative intensity and appearance of the characteristic reflections varying with composition. In the polymer-rich 25% bentonite–75% PVA formulation, the bentonite-associated reflections were relatively weak and broad, while the PVA contribution was more prominent. These changes are consistent with a reduced relative contribution of the clay phase and possible changes in the structural organization of bentonite in the PVA-rich matrix. Although such broadening and intensity changes may be compatible with modified clay stacking or polymer–clay interactions, they do not provide conclusive evidence of PVA intercalation into the bentonite galleries. Increasing the total solids content resulted in more pronounced diffraction features, consistent with the greater contribution of the solid phase.

The 50% PVA–50% bentonite formulation exhibited diffraction features arising from both PVA and bentonite, with the bentonite-associated reflection appearing relatively weak and broad. These features indicate changes in the structural organization of both phases compared with the individual components and are consistent with interactions between PVA and bentonite. The observed diffraction pattern may be compatible with partial modification of clay stacking and polymer–clay association; however, confirmation of an intercalated or exfoliated morphology would require complementary techniques such as TEM or SAXS. Increasing the solids content increased the overall diffraction intensity while retaining the characteristic contributions of both phases. Taken together with the drying and morphological results, the XRD observations suggest that the 50:50 formulation provides a favorable balance between the polymer and clay phases under the conditions investigated. However, the present results do not establish that this composition possesses a definitively optimized or intercalated microstructure.

When a system contains a lot of clay (75% bentonite–25% PVA), the most common diffraction patterns are sharp and strong basal reflections. This is especially true when the solids content is 10%. The changes in the basal (001) reflection of montmorillonite (2θ ≈ 6–8°) and the semi-crystalline (101) reflection of PVA (2θ ≈ 19–20°) provide information on composition-dependent changes in the structural organization of the PVA–bentonite composite coatings. The relative intensity of the characteristic bentonite diffraction peak decreased with increasing PVA fraction, particularly in the PVA-rich and 50:50 formulations. In addition, the PVA-associated crystalline reflection became weaker and broader in the composite coatings, indicating changes in the crystalline ordering of PVA in the presence of bentonite. These changes may be associated with interactions between PVA chains and clay surfaces that influence polymer-chain packing. However, the XRD results alone cannot establish the specific nature of these interactions or the extent of polymer intercalation within the clay galleries. In the clay-rich formulations, the more pronounced basal reflection indicates a greater contribution from the ordered layered structure of bentonite. Increasing the total solids content also increased the overall diffraction intensity, which may be related to the higher concentration and packing of the solid phases.

Overall, the XRD results indicate that both the PVA to bentonite ratio and total solids content influence the structural organization of the composite coatings. The altered diffraction features observed for the 50:50 formulation, together with its drying and morphological characteristics, suggest a favorable balance between the polymer and clay phases under the investigated conditions. However, these observations should not be interpreted as direct confirmation of an intercalated morphology or suppression of tactoid restacking. The observed composition-dependent structural changes are consistent with those reported for PVA–montmorillonite systems in the literature [33,34,44,50,51].

### 3.6. SEM Analysis of Bentonite–PVA Coatings

Scanning electron microscopy (SEM) was used to observe the morphology of the prepared coatings. At lower bentonite contents, the structural characteristics were predominantly influenced by the PVA phase, whereas at an approximately equal PVA to bentonite ratio, contributions from both polymer and clay phases became evident. This behavior may reflect the combined influence of polymer–clay interactions, clay particle associations, and changes in polymer-chain mobility during film formation and drying.

**Figure 5:**
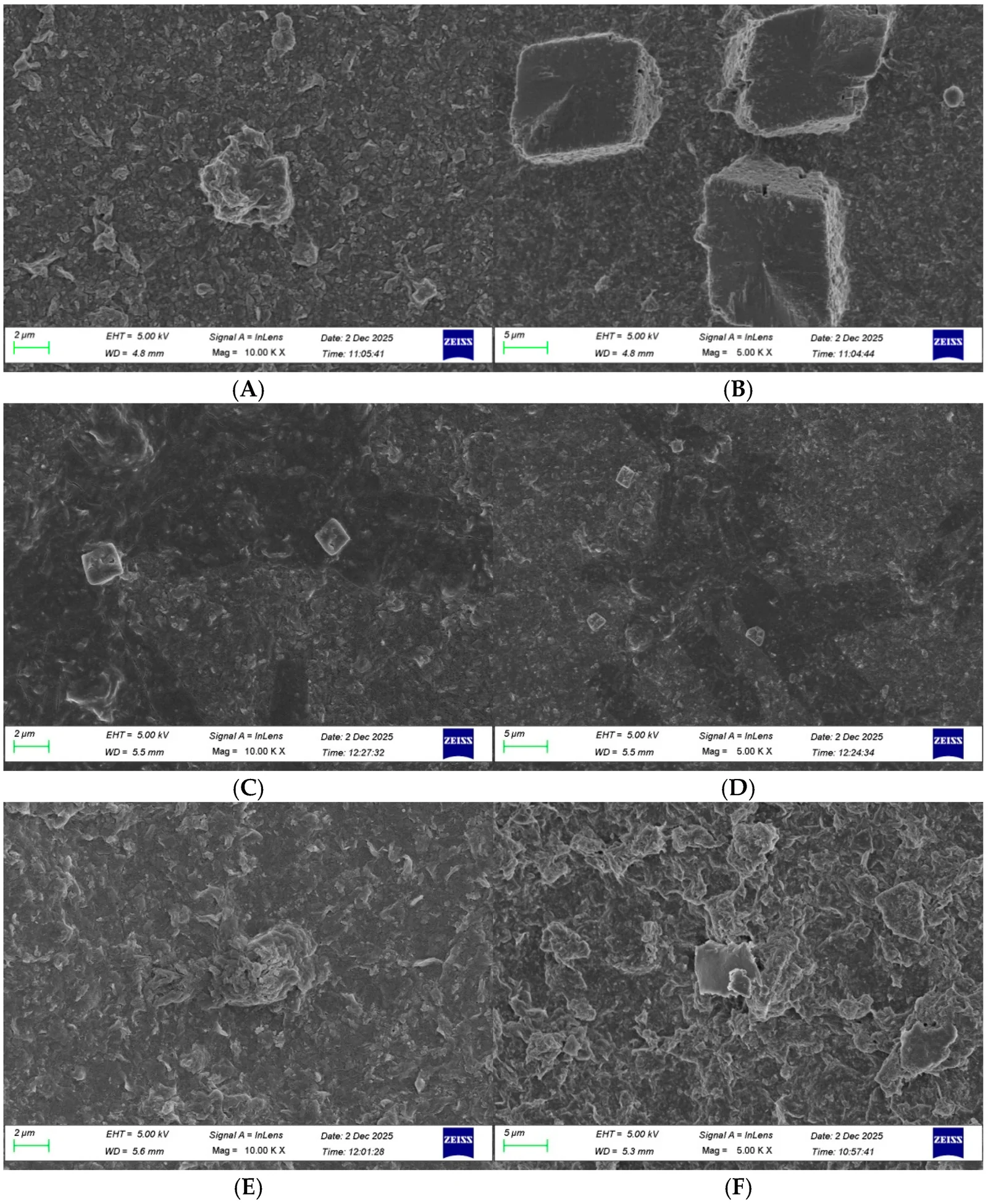
SEM micrographs for 25% bentonite–75% PVA (5% solids) coating at 2 micron scale (**A**), SEM micrographs for 25% bentonite–75% PVA (5% solids) coating at 5 micron scale (**B**), SEM micrographs for 25% bentonite–75% PVA (10% solids) coating at 2 micron scale (**C**), SEM micrographs for 25% bentonite–75% PVA (10% solids) coating at 5 micron scale (**D**), SEM micrographs for 50% bentonite–50% PVA (10% solids) coating at 2 micron scale (**E**), SEM micrographs for 50% bentonite–50% PVA (10% solids) coating at 5 micron scale (**F**).

The 25% bentonite–75% PVA coating exhibited a relatively uniform microstructure, with morphological characteristics predominantly governed by the PVA-rich matrix. Figure 5A,B show that the coating surface is smooth, solid, and has no microcracks, voids, or particle pull-out at the 2 µm scale, despite the high solids content. The bentonite platelets do not look like separate groups of particles, suggesting that they are either spread out on their own or exist as very small tactoids surrounded by the PVA matrix. Some of the shapes near the surface are angular and composed of single clay platelets that maintain their platelet shape and adhere tightly to the polymer around them.

Figure 5C,D show that the coating maintains a smooth surface, even over bigger areas at the 5 µm scale. There are no signs of phase separation, filler segregation, or aggregation on the micron scale, as shown in the SEM images. To further endorse this finding, a detailed phase separation study was performed using rigorous morphological and EDS analyses at each and every micron position. The surface exhibited smooth, low-amplitude undulations without pronounced rough features. These morphological variations may be associated with structural rearrangements during solvent evaporation and film consolidation. However, the SEM observations alone cannot directly establish the underlying polymer-chain rearrangement mechanism. There are no gaps or exposed clay domains between the PVA and bentonite, which makes them adhere tightly. This is because a lot of hydrogen bonds form between the hydroxyl groups of PVA and the edges and basal faces of the clay platelets.

The SEM pictures show that PVA effectively changes the surface of the material and acts as a good binder. There is a lot of polymer coverage, which keeps the clay particles from adhering to other clay particles and enhances steric stabilization. This means that the structure is mostly composed of the polymer. This microstructure should give the coating strength, flexibility, and drying uniformity, making it a good choice for coatings that are both protective and useful.

The microstructure of the coating with 50% bentonite and 50% PVA was very different. There are both organic and inorganic parts in the coating that function together. At the 2 µm scale (Figure 5E), the surface is thick and particles self-aggregate, but the surface becomes a lot rougher. Localized agglomerated features and structures that resemble tactoids are responsible for this phenomenon. These are composed of bentonite platelets that are stacked or partially peeled off. These observations indicate that the polymer can only completely cover all clay surfaces in certain areas when the quantities of polymer and clay are equivalent. This means that the clay particles can keep interacting with other clay particles.

Despite the presence of inorganic clusters, no obvious interfacial voids, cracks, or particle pull-out were observed in the examined SEM regions, indicating good morphological continuity between the bentonite-containing regions and the surrounding PVA matrix. The absence of pronounced interfacial defects is consistent with favorable compatibility between the two phases. Interactions between PVA and bentonite may contribute to this morphology; however, the SEM observations alone cannot establish the nature or strength of these interfacial interactions.

Figure 5F shows the microstructure at the 5 µm scale. The polymeric binder holds the bentonite platelets and aggregates together, forming a framework that is not organic and is only partially continuous. Because there is a high solids content, the polymers cannot move around as much. If there were a lot of polymer in the mixture, the surface would be smoother. At this point, PVA mostly fills the gaps between clay clusters instead of making a smooth, continuous film.

The SEM results show that adding more bentonite always changes the structure of the coating in the same way. It evolves from being smooth and composed mostly of the polymer to one that is composed of both the polymer and clay. Even when there is more clay in the mix, the PVA still functions well as a binder and interfacial modifier. However, the clay particles self-aggregate more and the surface is rougher, diminishing steric stabilization.

The coating composed of 50% bentonite and 50% PVA has a mixed microstructure that should make it stiffer, better at blocking things, and last longer. This is due to the inorganic framework being only partially open. However, this means that the surface is not as smooth and flexible as it would be at a high polymer content. The SEM results are as expected based on the swelling. These results show the importance of the composition and interaction of polymers and clay in the microstructure and performance of the coating.

The SEM micrographs illustrate the interplay between polymer adsorption on clay surfaces, clay–clay attractive interactions, and the kinetic constraints imposed by solvent evaporation during film formation. The smooth, crack-free surface of coatings with a high PVA content (25% bentonite and 75% PVA) shows no signs of platelet pull-out, which is what is expected from polymer-bridging adsorption and steric stabilization of individual platelets and small tactoids. This is because the adsorbed PVA layers keep montmorillonite from stacking face-to-face and help it to spread out evenly at the micron level. The absence of interfacial debonding characteristics indicates robust interfacial adhesion. This is mostly because the hydroxyl groups on PVA and the basal and edge –OH sites on clay platelets are held together by hydrogen bonds. When the filler content increases in the 50% bentonite–50% PVA system, the polymer does not cover the clay’s surface enough, which allows the platelets to partially re-aggregate through van der Waals and electrostatic interactions. This is why localized tactoid-like features and increased roughness are observed. It is important to remember that cohesive interfaces stay together without gaps or pull-outs. This means that polymer adsorption is still strong enough to maintain load transfer across the polymer–clay interfaces, even in areas where the polymer is grouped together. In regions where solvent transport may be restricted, the observed clay-rich domains appear to form a more extended and partially continuous inorganic network within the polymer matrix. When the amount of solids increases, and the polymer’s ability to move around decreases, and structures are trapped in a non-equilibrium state. There are many examples of transitions in polymer–montmorillonite systems that depend on composition. For example, smooth films with a high polymer content can turn into mixed organic–inorganic frameworks. These changes in the interfacial area and tortuosity of transport pathways have a direct impact on stiffness, barrier performance, and drying uniformity [33,34,44,50,51,52,53,54].

The elemental analysis results of pure PVA and bentonite are compiled in Table 1 based on the EDS results.

### 3.7. Drying of 5% Solids–95% Water Coatings

**Figure 6:**
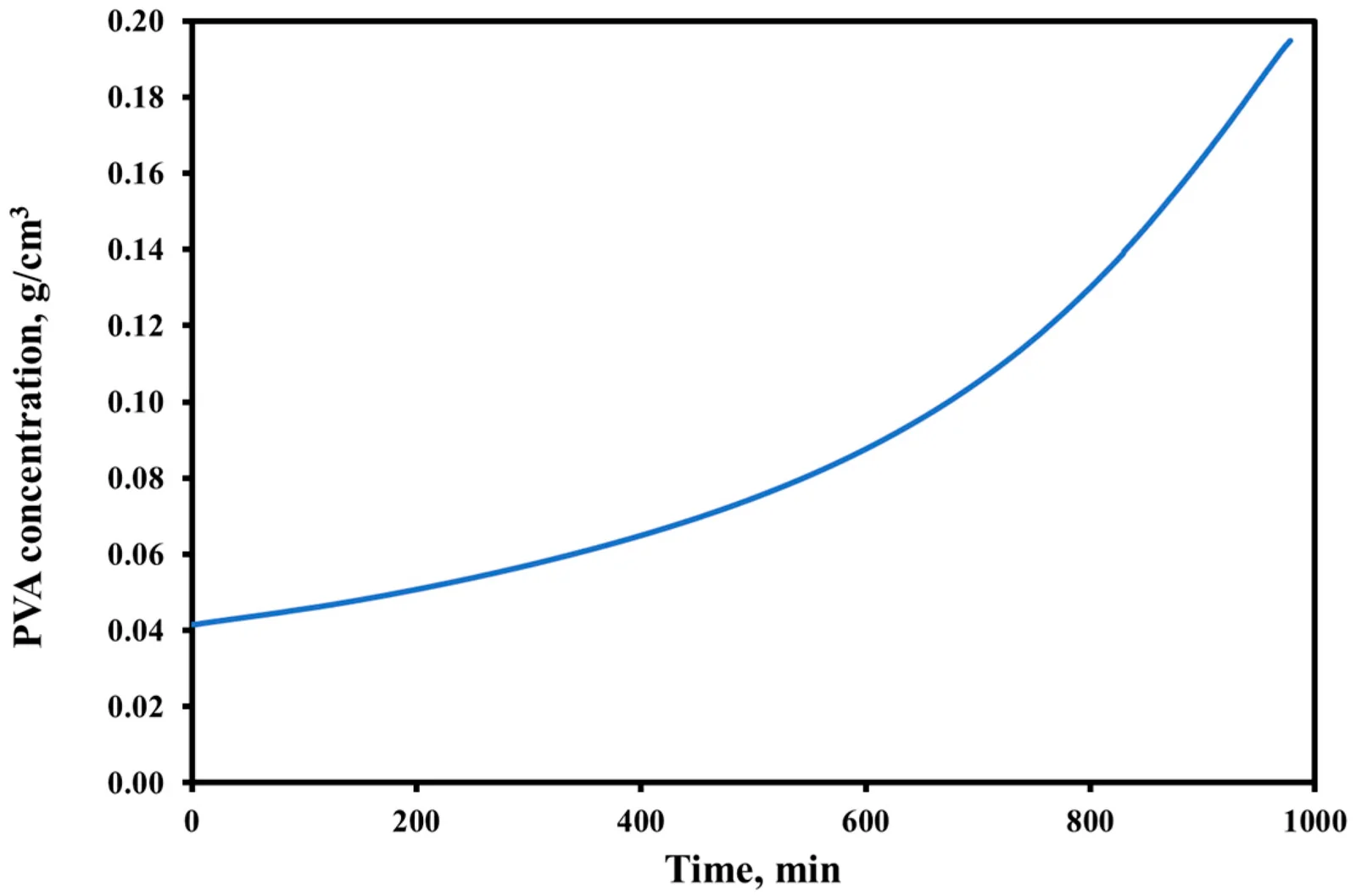
PVA concentration as a function of time in a coating with 5% pure PVA.

**Figure 7:**
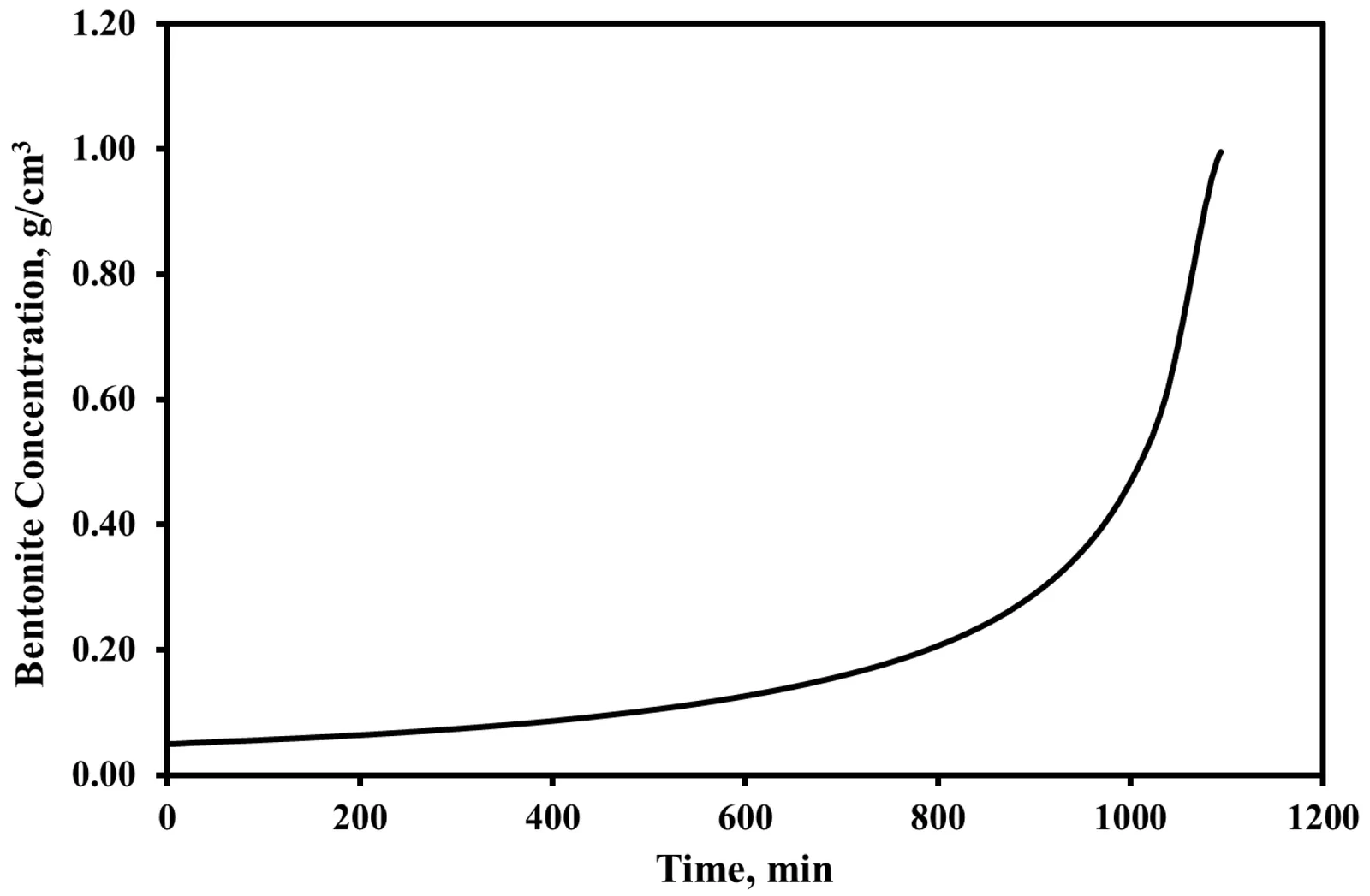
Bentonite concentration as a function of time in a coating with 5% pure bentonite.

**Figure 8:**
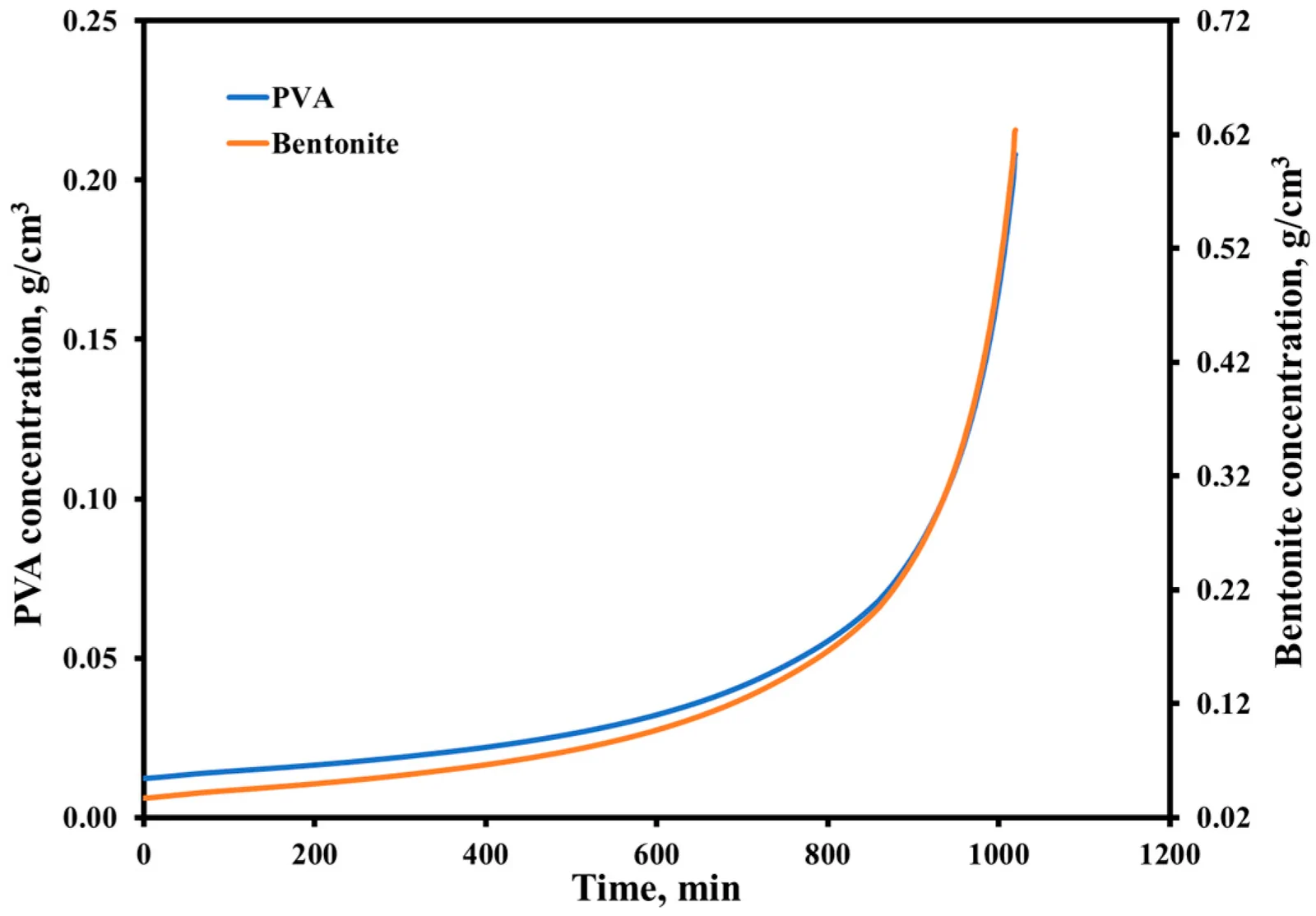
PVA and bentonite concentrations as a function of time in a coating with 5% solids (25% PVA + 75% Bentonite).

**Figure 9:**
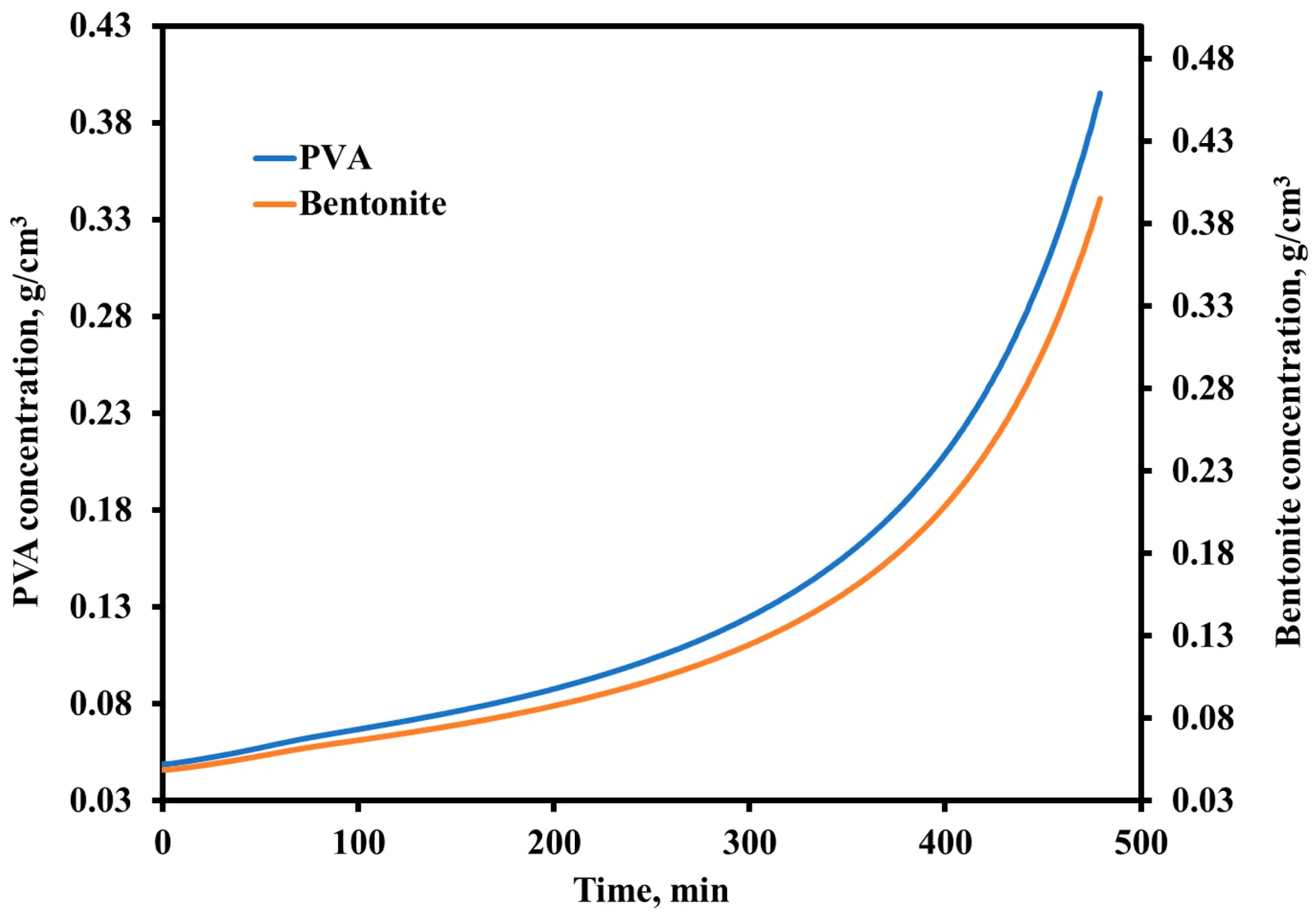
PVA and bentonite concentrations as a function of time in a coating with 5% solids (50% PVA + 50% Bentonite).

**Figure 10:**
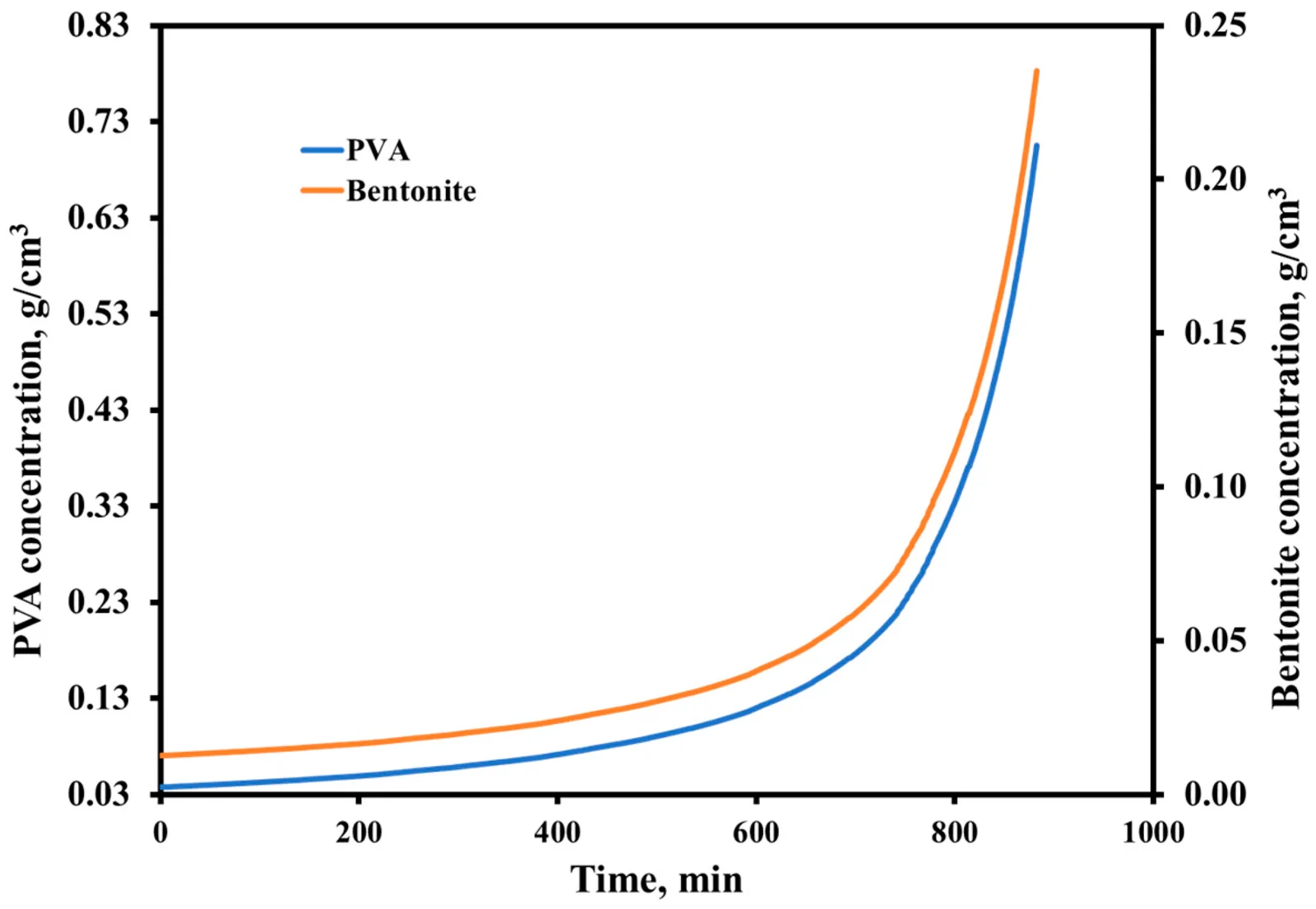
PVA and bentonite concentrations as a function of time in a coating with 5% solids (75% PVA + 25% Bentonite).

**Figure 11:**
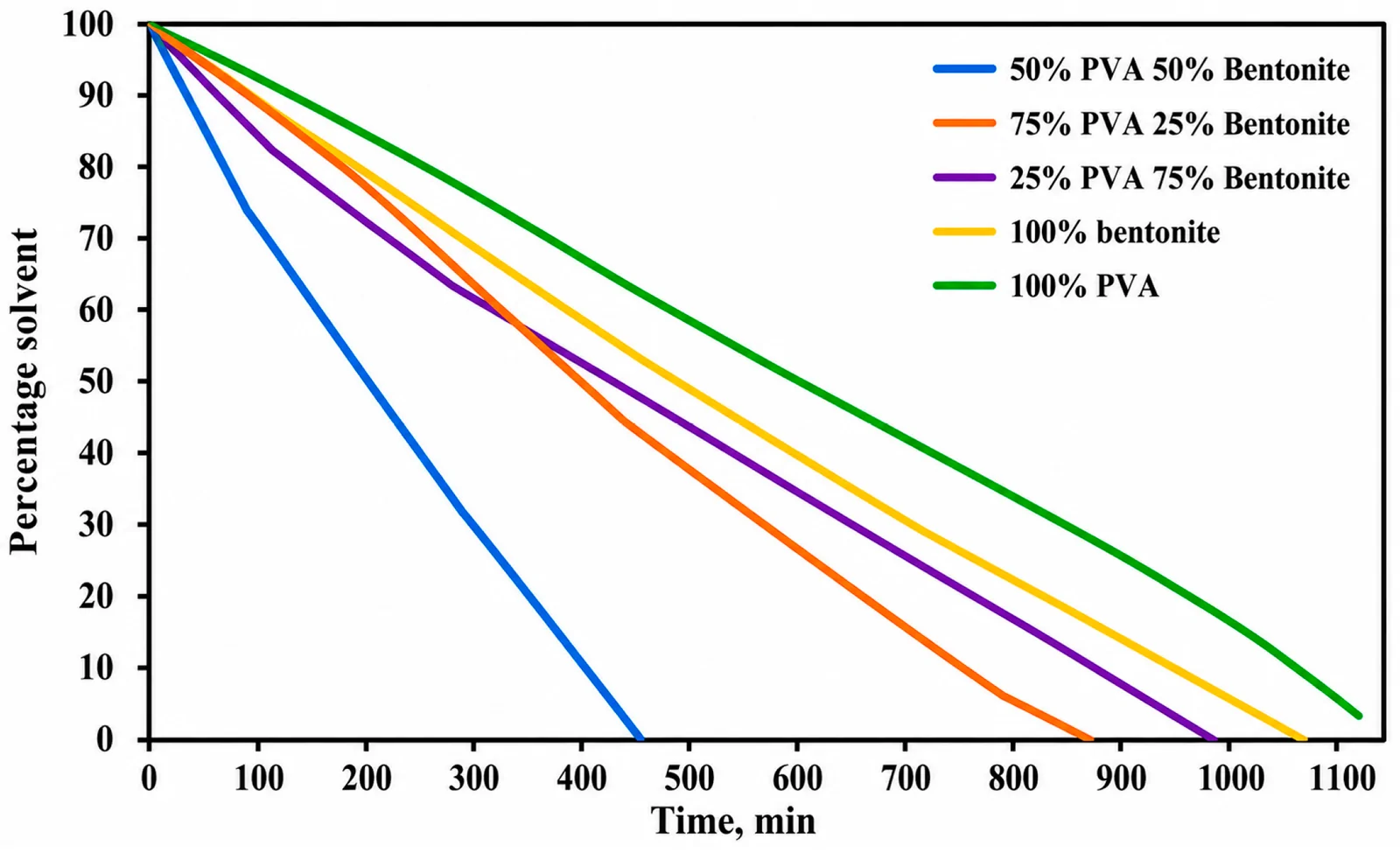
Residual solvent as a function of time for various coatings with 5% solids.

**Figure 12:**
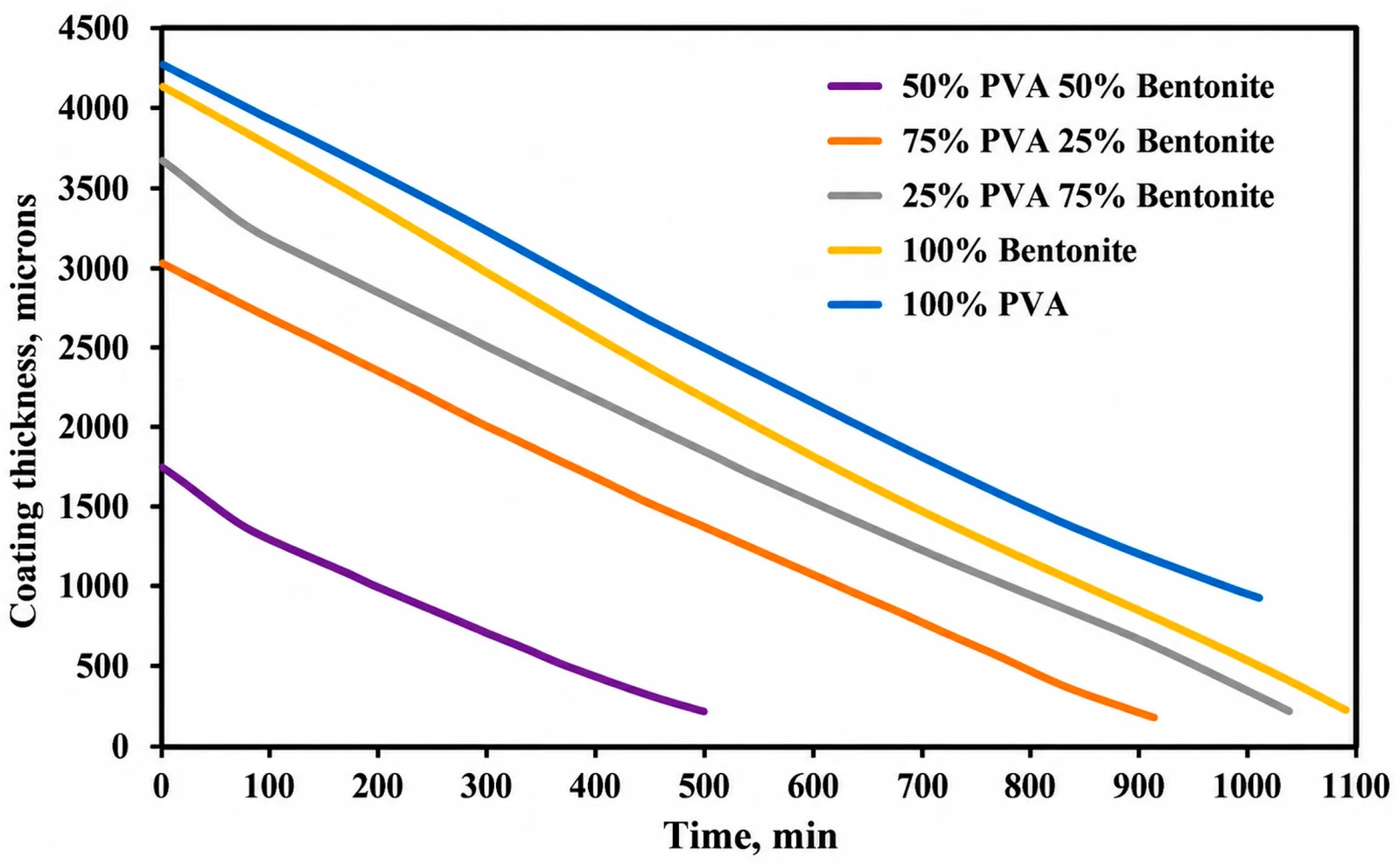
Coating thickness as a function of time for coatings with 5% solids.

Figure 6, Figure 7, Figure 8, Figure 9, Figure 10, Figure 11 and Figure 12 compare the drying behaviors of the 5 wt.% PVA–bentonite coatings as a function of time with different compositions. Across all formulations, progressive solvent loss resulted in an increase in the concentrations of the non-volatile PVA and bentonite phases and a corresponding decrease in coating thickness. The drying profiles generally exhibited an initial period dominated by relatively rapid solvent loss, followed by a slower stage as the coatings became increasingly concentrated. The transition between these regimes depended strongly on the PVA to bentonite ratio. Increasing the bentonite fraction was associated with an earlier transition toward slower solvent removal, whereas PVA-rich formulations exhibited a comparatively prolonged drying period. These differences demonstrate that the coating composition influences solvent removal and film consolidation even when the initial total solids content is maintained at 5 wt.%.

Among the investigated compositions, the 50% PVA–50% bentonite formulation exhibited the most favorable overall drying response, reaching equilibrium in approximately 480 min. By comparison, the equilibrium drying times were approximately 978 min for pure PVA, 880 min for the 75% PVA–25% bentonite formulation, and 600 min for the 25:75 formulation. The final thickness also varied considerably with composition. For example, the 75:25 and 25:75 coatings exhibited thickness reductions from approximately 3042 to 481 µm and 1800 to 716 µm, respectively. Overall, the results indicate that neither a strongly polymer-rich nor a strongly clay-rich composition provided the most favorable drying behavior. Instead, the 50:50 formulation provided a comparatively balanced response between solvent removal and film consolidation under the investigated conditions [55].

Table 2 displays the results of a comparative drying analysis of five coating systems. These systems include pure PVA, pure bentonite, and mixed compositions (75/25, 50/50, and 25/75 PVA to bentonite). The results show that the solvent evaporation behavior, concentration buildup, and film shrinkage develop as a function of composition and time.

### 3.8. Drying of 10% Solid Coatings of Various PVA–Bentonite Mixtures

**Figure 13:**
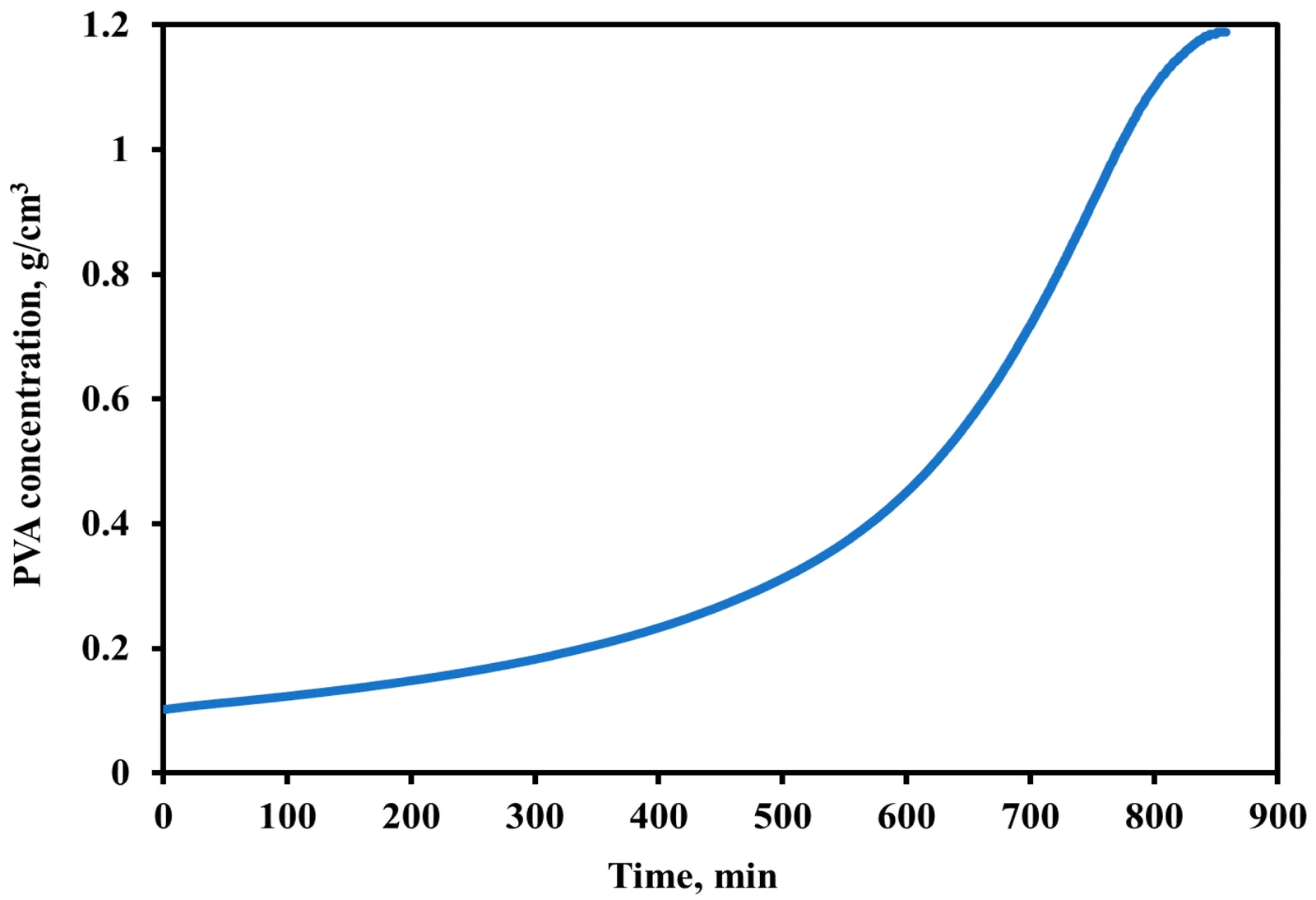
PVA concentration as a function of time in a coating with 10% pure PVA.

**Figure 14:**
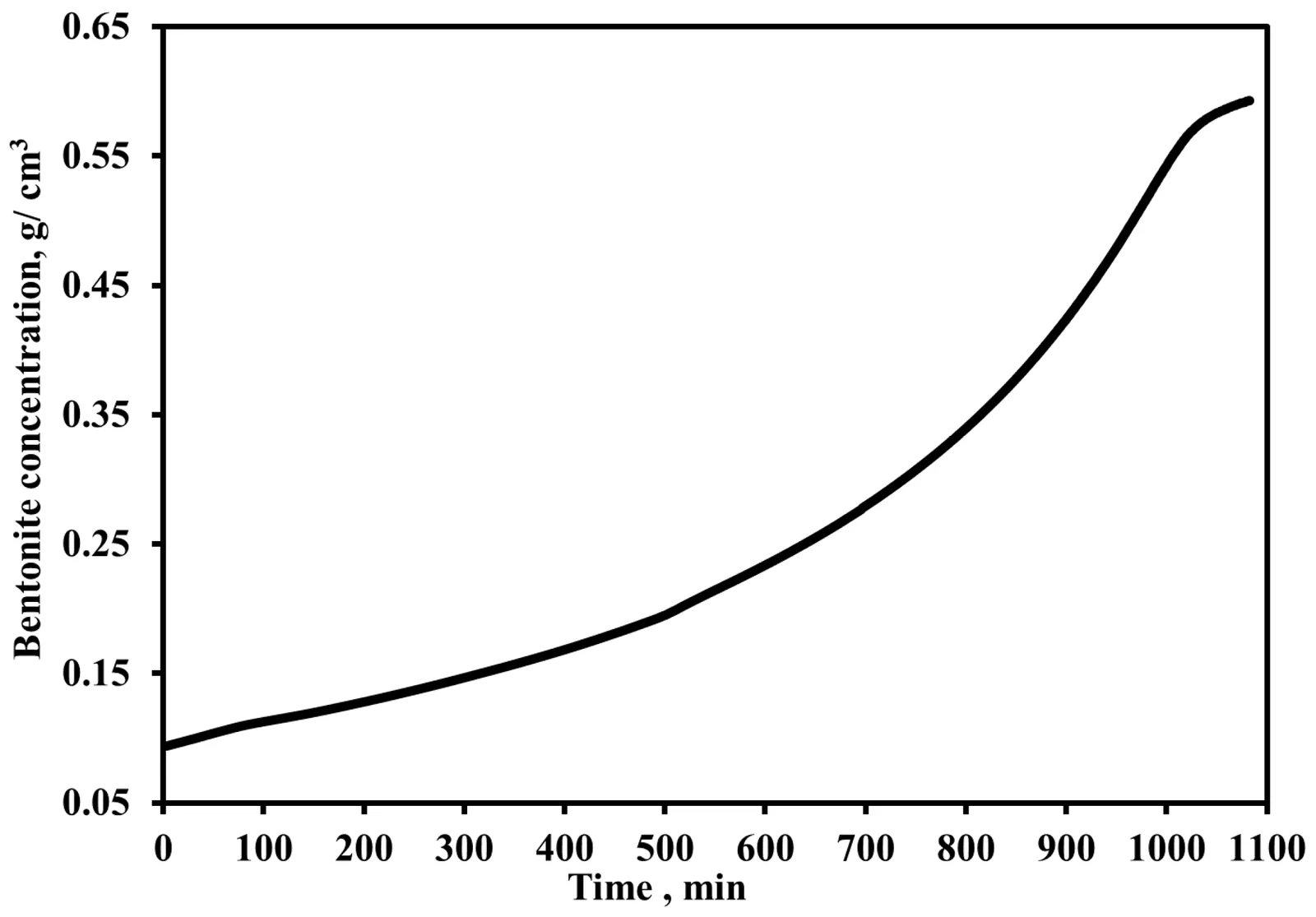
Bentonite concentration as a function of time in a coating with 10% pure bentonite.

**Figure 15:**
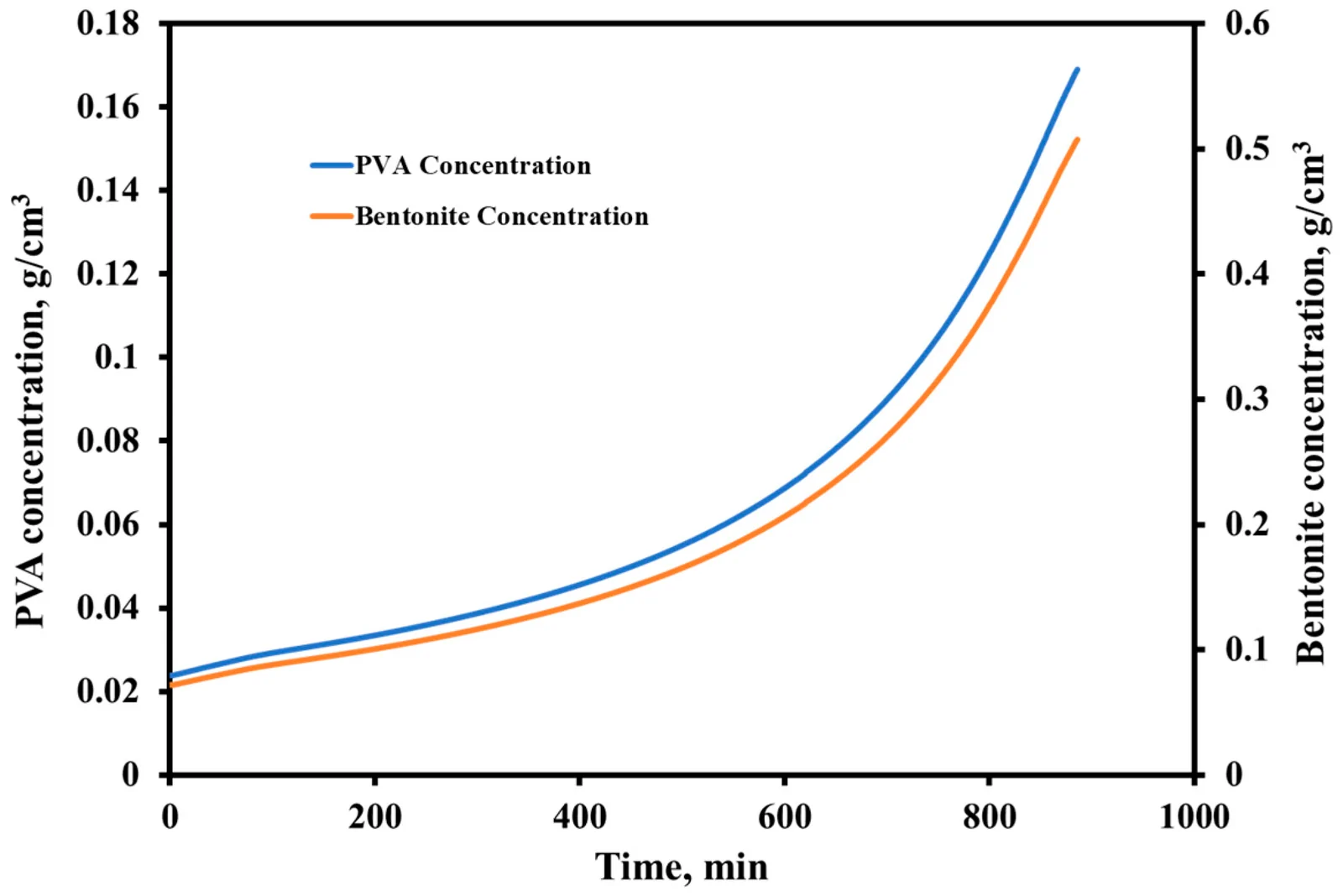
PVA and bentonite concentrations as a function of time in a coating with 10% solids (25% PVA + 75% bentonite).

**Figure 16:**
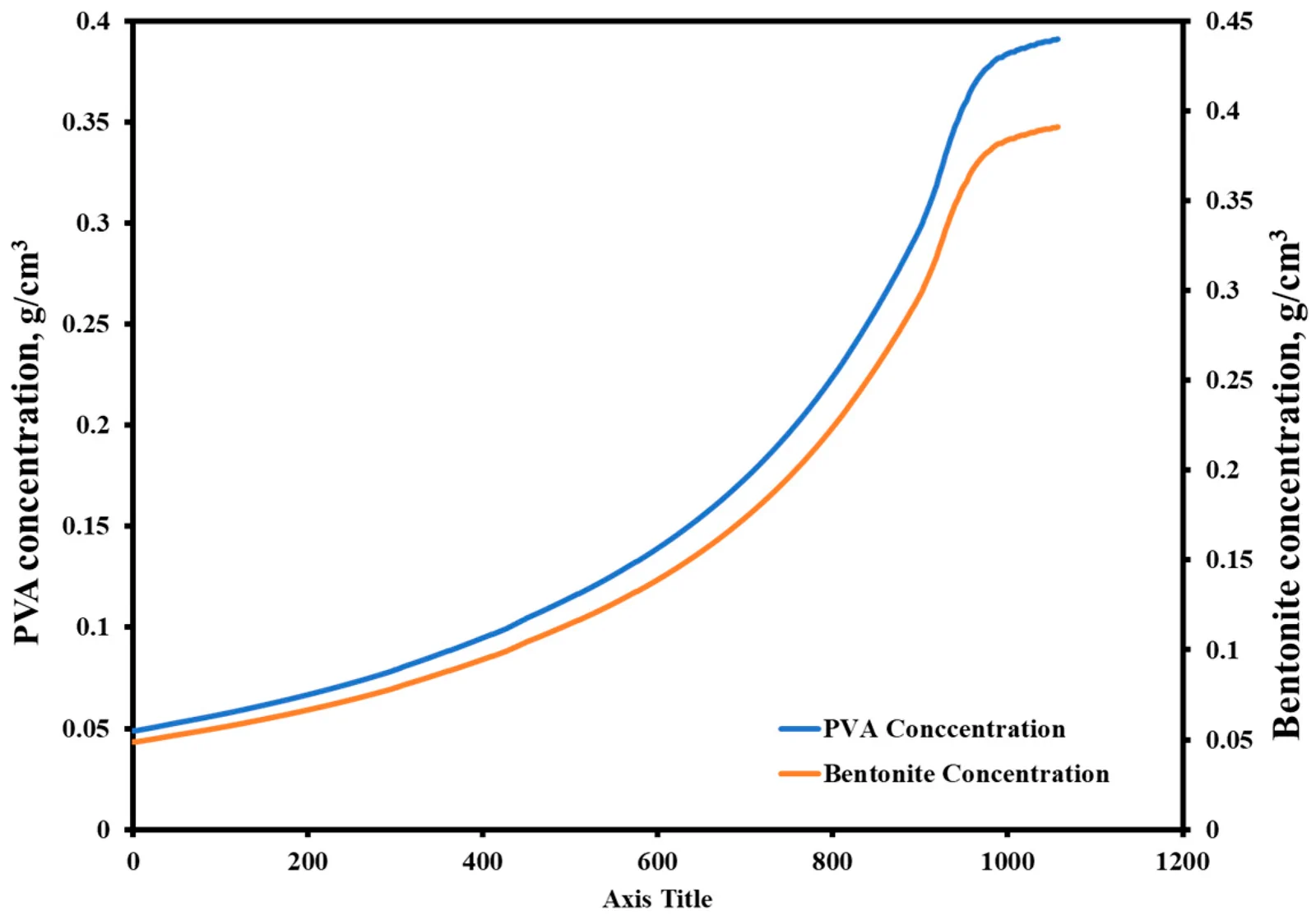
PVA and bentonite concentrations as a function of time in a coating with 10% solids (50% PVA + 50% bentonite).

**Figure 17:**
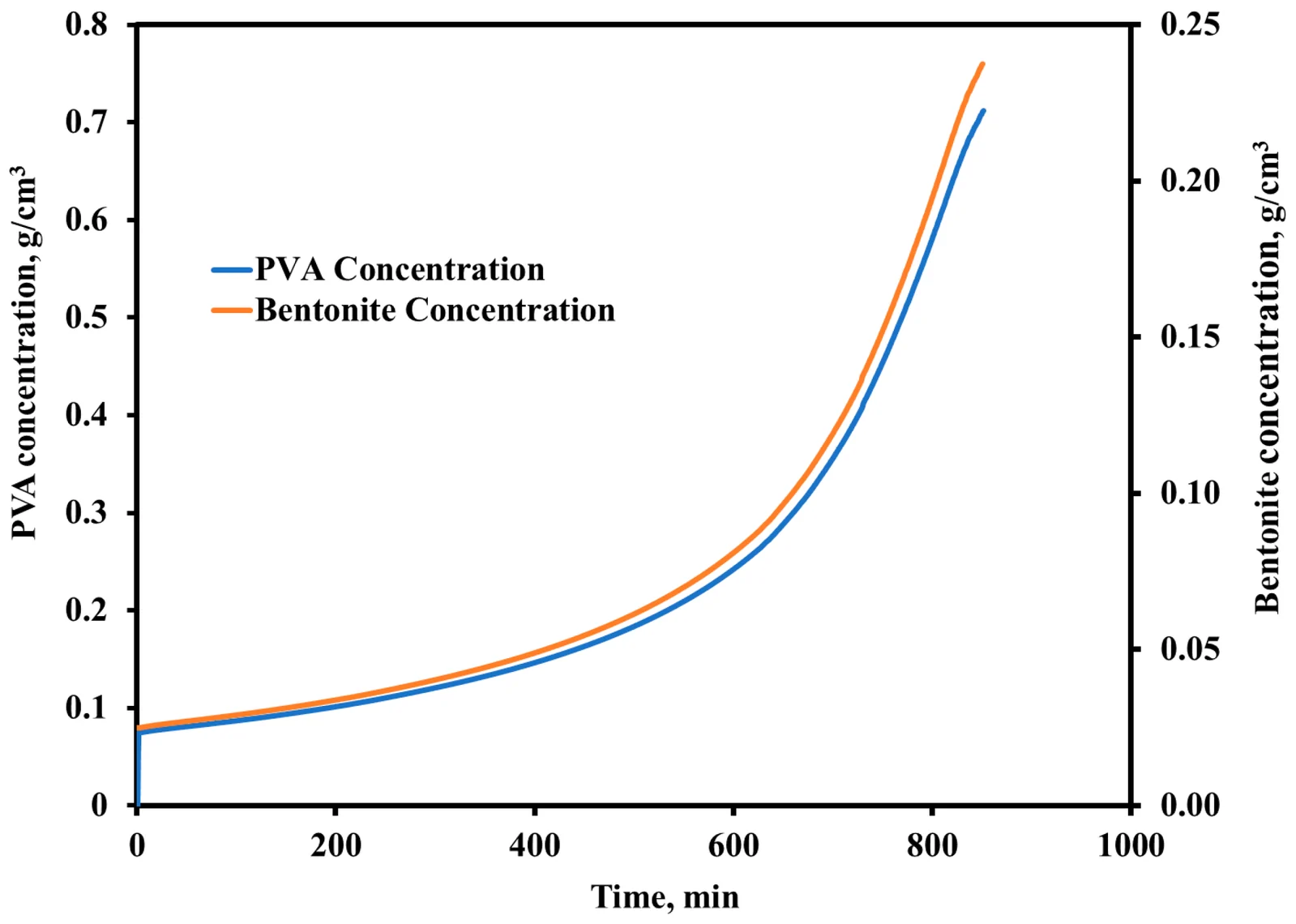
PVA and bentonite concentrations as a function of time in a coating with 10% solids (75% PVA + 25% bentonite).

**Figure 18:**
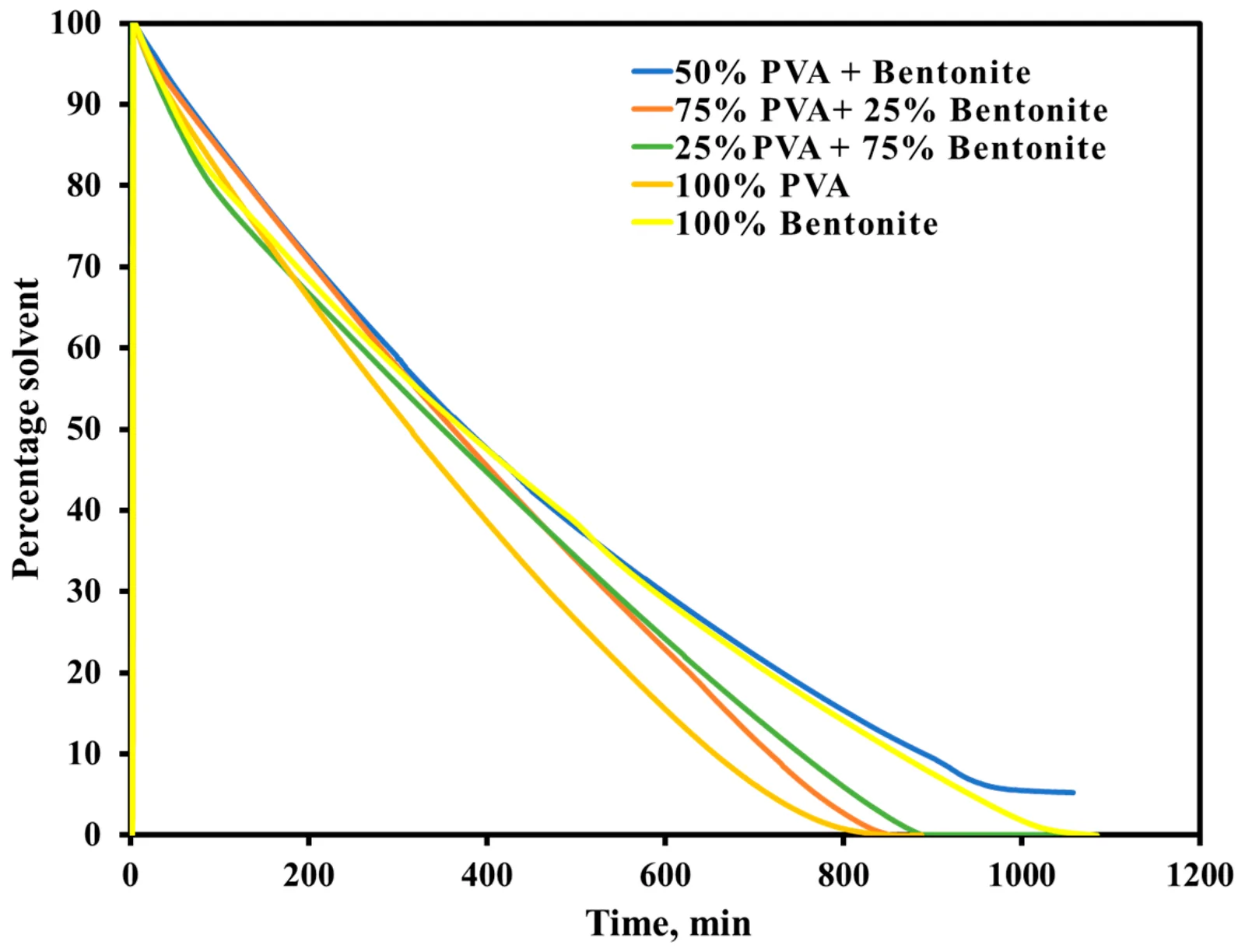
Residual solvent as a function of time in 10% solution coatings of various mixtures.

**Figure 19:**
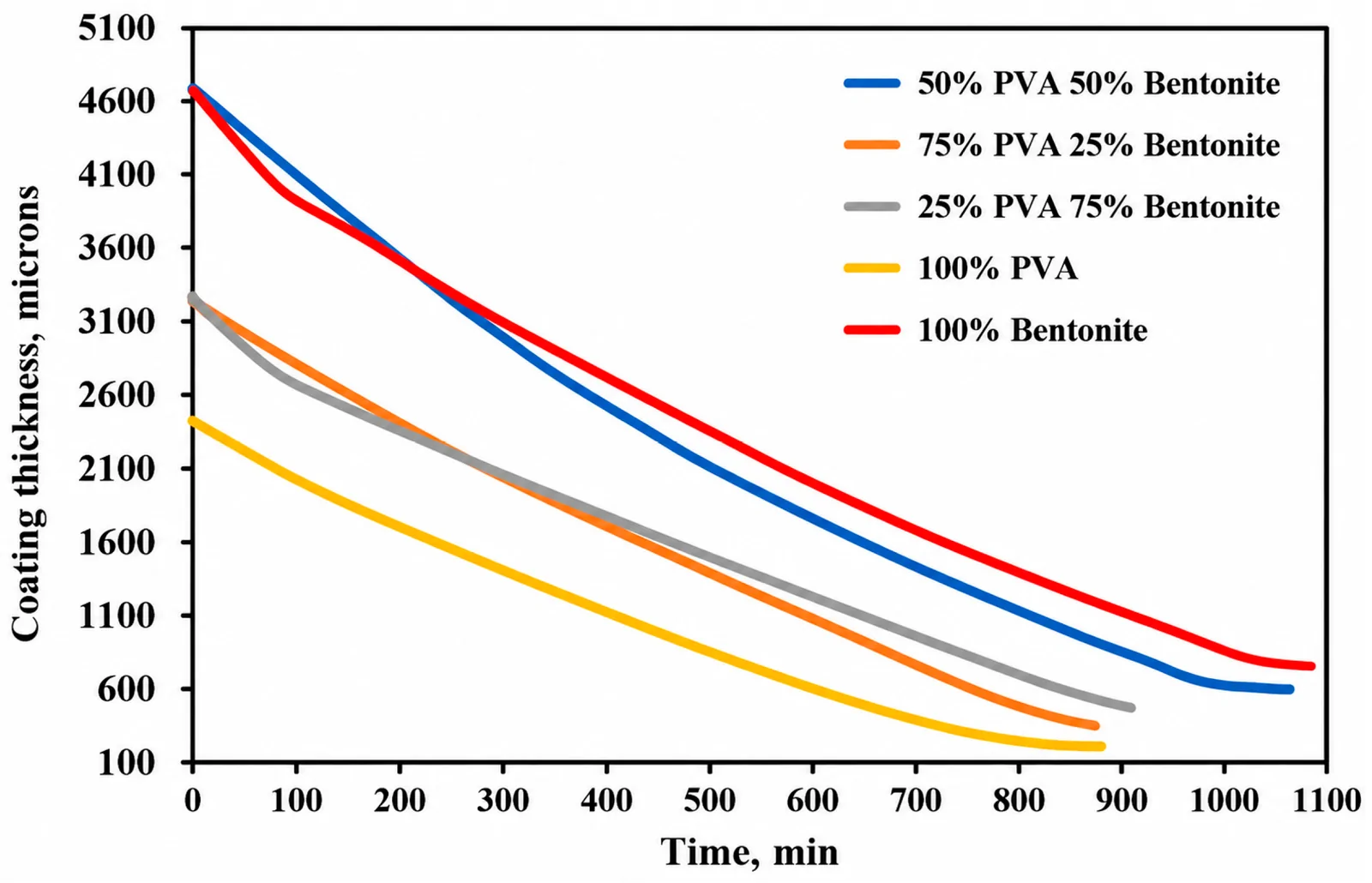
Coating thickness as a function of time in 10% solution coatings of various mixtures.

The drying profiles of the 10 wt.% coatings showed a composition-dependent increase in PVA and bentonite concentrations with time, accompanied by progressive solvent loss and reduction in coating thickness (Figure 13, Figure 14, Figure 15, Figure 16, Figure 17, Figure 18 and Figure 19). In general, the coatings showed an initial period of relatively rapid solvent removal followed by slower drying as the solids became increasingly concentrated. The extent and duration of these changes varied with the PVA to bentonite ratio. PVA-rich coatings exhibited comparatively prolonged drying, while increasing the bentonite fraction altered the rate of solvent loss and resulted in greater residual solvent in some formulations. For example, the 25% PVA–75% bentonite coating retained approximately 30% solvent during the later drying stage, whereas the 75:25 formulation approached an equilibrium residual solvent content of approximately 10–12%.

Among the 10 wt.% formulations, the 50% PVA–50% bentonite coating reached equilibrium most rapidly, with a total drying time of approximately 480 min, compared to approximately 880 min for pure PVA, 1083 min for pure bentonite, 881 min for the 75:25 formulation, and 600 min for the 25:75 formulation. All formulations also exhibited substantial thickness reduction during drying, confirming progressive coating consolidation as solvent was removed. Overall, the results demonstrate that both the drying time and dimensional changes of the 10 wt.% coatings were strongly dependent on the PVA–bentonite composition, with the 50:50 formulation showing the shortest drying time among the compositions investigated.

Table 3 summarizes the drying transitions, composition ratios, solvent retention, coating thickness, and total drying time for the PVA–bentonite coatings. The drying kinetics and film densification depend strongly on composition, governed by polymer–filler interactions and diffusion pathways. Pure PVA dried in approximately 880 min, and pure bentonite in 1083 min, whereas blends of 75:25, 50:50, and 25:75 PVA–bentonite required 881, 480, and 600 min, respectively, demonstrating that the 50:50 mixture dried most rapidly due to its optimized microstructure. Solvent retention augmented with increased clay loading, attaining 30% for the 25:75 blend, as bentonite’s stratified structure confined moisture and impeded diffusion. The film thickness diminished significantly for the 50:50 mixture, indicating optimal compaction and densification. The drying transition transpired at around 270 min for pure PVA, 700 min for pure bentonite, and 360 min for the 50:50 blend, indicating optimal diffusion control in the balanced formulation. Pure PVA films exhibited smoothness, pure bentonite films showed brittleness, and the 50:50 blend produced homogenous, compact coatings with negligible flaws. In summary, the inclusion of moderate amounts of bentonite enhanced the drying rate, solvent diffusion, and film uniformity, establishing the 50:50 PVA to bentonite ratio as the most effective and structurally stable formulation.

### 3.9. Rheological Analysis of Bentonite–PVA Dispersions

Previous studies by İşçi et al. [56] and Brandenburg and Lagaly [57] have reported composition-dependent rheological behavior in PVA–bentonite/montmorillonite systems. Consistent with the composition-dependent behavior reported in these studies, the present results showed that the 50% PVA–50% bentonite formulation exhibited comparatively favorable film-forming characteristics among the formulations investigated. Therefore, the rheological analysis of the 50% bentonite–50% PVA solution is presented in this section. However, a detailed thixotropic analysis of all dispersions is reported in the next section.

**Figure 20:**
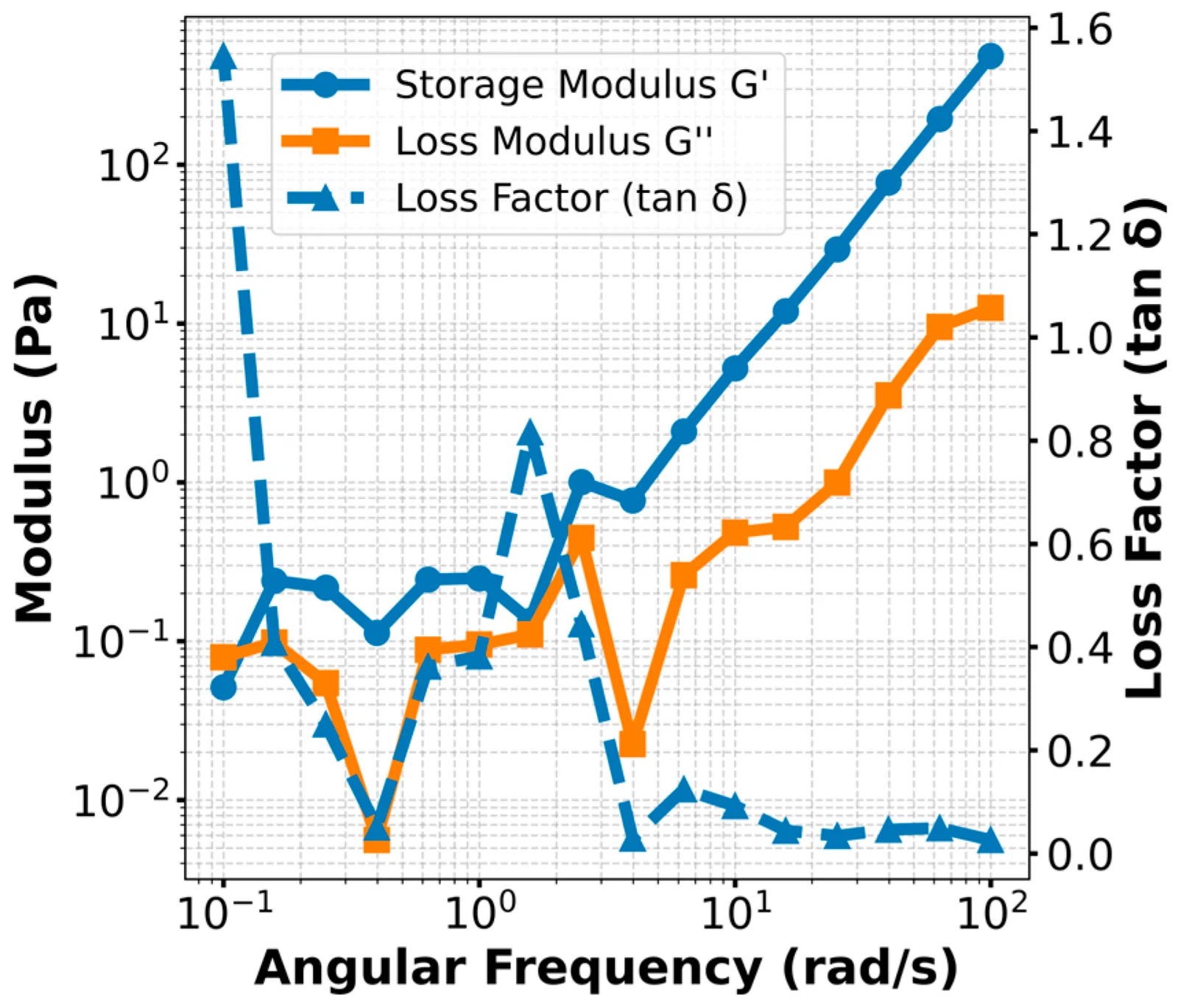
Frequency-dependent viscoelastic behavior of 50% bentonite–50% PVA with 10% solids dispersion.

Figure 20 shows the stress response of the 50% bentonite–50% poly(vinyl alcohol) (PVA) dispersion with 10 wt.% total solids, illustrating the oscillation changes as a function of time. How often these changes occur determines the stress response. The loss modulus (G″), the loss factor (tan δ), and the storage modulus (G″) all change when the angular frequency (ω) changes. The polymer–clay network has the same thickness and stretch.

We investigated angular frequencies from 0.1 to 100 rad s^−1^ and found that the storage modulus (G′) is usually higher than the loss modulus (G″). The dispersion could be either more solid or more flexible. This response stems from the formation of robust hydrogen bonds between the hydroxyl groups in PVA and the silanol groups on the surfaces of the bentonite platelets. This created a web of three-dimensional spaces that were linked together. At equivalent mass fractions, the bentonite platelets make it harder for the polymer chains to move, shifting the system to elastic energy storage.

An increase in the angular frequency increases G′ and G″. This means that the molecules do not have as much time to cool down when timescales are shorter. When the angular frequency is high, the connections between the clay and the polymer and between polymer chains cannot quickly return to their normal shapes, driving elastic dominance. This behavior is characteristic of polymer–clay mixtures with fillers that are evenly spread out and function well together.

If the angular frequency is less than 1 rad s^−1^, G′ decreases sharply and G″ increases slightly. The dispersion loses more energy because it has been relaxing for a long time and is changing shape. At the lowest frequencies, the loss factor (tan δ) rises quickly, reaching above 1. This behavior indicates that major changes are occurring. The weak physical network might break or change shape slightly when it bends slowly, allowing the polymer chains and clay tactoids to move in different directions.

The tan δ is highest at one frequency. This allows the clay and polymer to function together. Tan δ is less than 1 when the angular frequency is higher than 10 rad s^−1^. This means that the network is strong and can sustain dynamic mechanical loads without collapsing.

The 50% bentonite–50% PVA (10 wt.% solids) mixture functions as a viscoelastic system that can change shape and move. It provides high structural resistance during rapid dynamic loading, while it transitions to a viscous fluid under slower, low-frequency shearing to control the flow of liquids. These features are advantageous for making coatings because they keep the coating’s mechanical integrity and make sure it flows well enough to spread evenly and form a film without any problems during processing.

**Figure 21:**
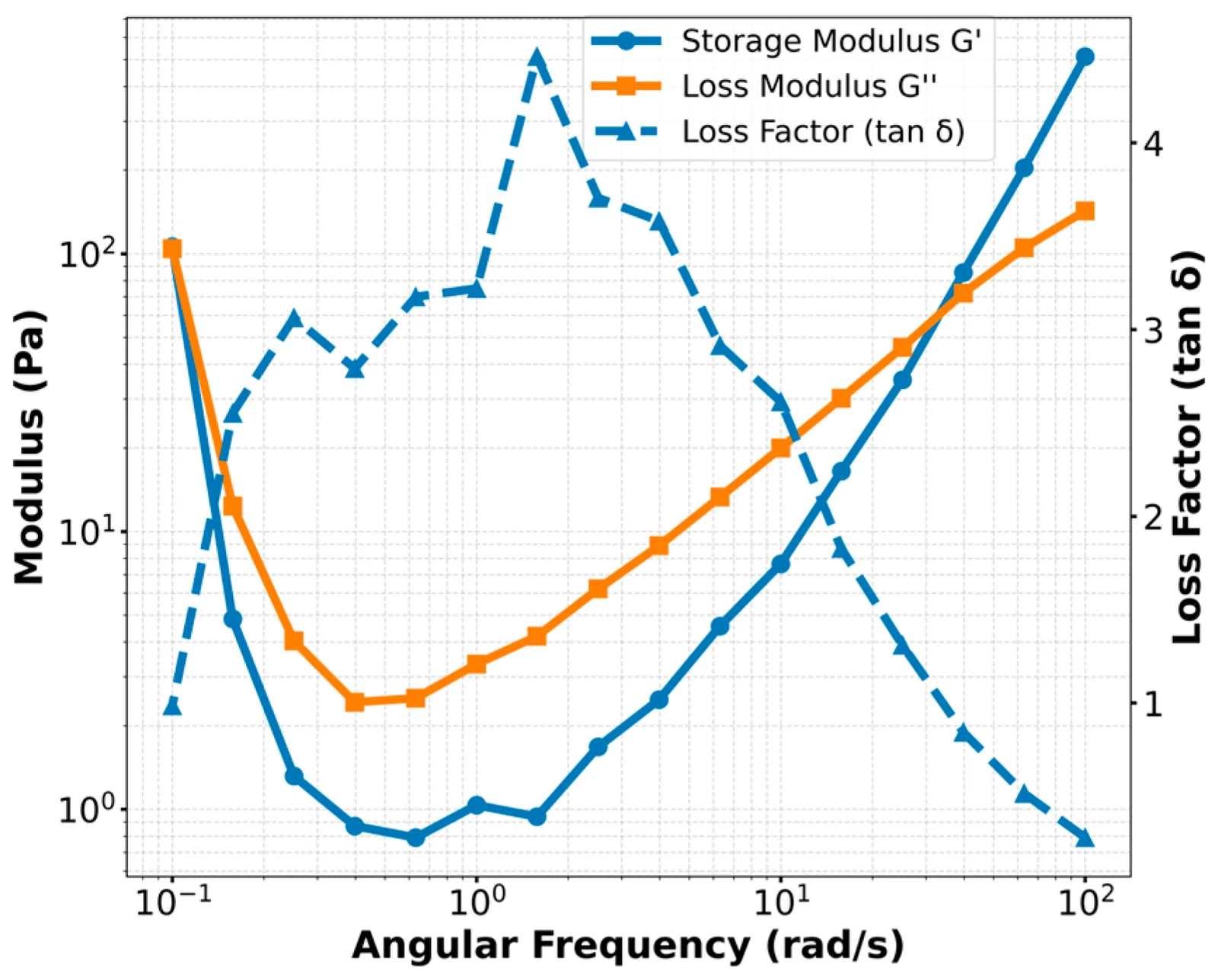
Frequency-dependent viscoelastic behavior of 10% pure PVA solution.

Figure 21 shows the storage modulus (G′), loss modulus (G″), and loss factor (tan δ) of the pure PVA solution (10 wt.% solids) as a function of frequency-sweep rheological response. The data show that pure PVA behaves very differently when it is pulled and stretched than the bentonite–PVA dispersion mentioned earlier.

In a wide range of middle frequencies (about 0.25–25 rad s^−1^), the loss modulus (G″) is larger than the storage modulus (G′). This means that the pure PVA solution mostly acts like a liquid or a thick liquid. This occurs in polymer solutions when chain entanglements mainly affect the rheological behavior, but no strong three-dimensional network forms. The G′ values are not very high at these frequencies. This means that molecules can move around easily, suppressing the storage of elastic energy.

When the frequency is lower (ω < 1 rad s^−1^), both G′ and G″ decrease. This is because bending the PVA chains loosens them and they slowly come apart. The loss factor (tan δ) is much higher than 1 (tan δ ≈ 3–4) in this regime. This means that the flow behavior dominates the viscoelastic response, and viscous dissipation is strong. This is in line with the fact that a polymer solution mostly responds by rearranging its chains instead of bouncing back.

As the frequency rises, both G′ and G″ rise sharply. This means that the time needed to relax becomes shorter and the resistance to bending becomes stronger. At high frequencies (ω ≥ 50 rad s^−1^), G′ and G″ are roughly equivalent, and tan δ is approximately one. This behavior shows the onset of elastic contributions because the chains are becoming tangled for a short time. This means that the polymer chains do not have enough time to relax while they are being deformed. However, the fact that there is no clear G′–G″ crossover at lower frequencies shows that pure PVA at 10 wt.% does not form a solid or gel-like network.

The pure PVA (10 wt.%) solution is mostly thick over a wide range of frequencies. Elasticity is only important for short periods of time. This is very different from the 50% bentonite–50% PVA dispersion, where strong interactions between the clay and the polymer make it very clear that the polymer is more elastic. Pure PVA is thick, which helps with leveling and flow when applying a coating. However, its low elastic strength shows that bentonite can help make composite coatings stronger and more stable.

The frequency-dependent viscoelastic response shows that there is a physically percolated polymer–clay network in the 50% bentonite–50% PVA dispersion. The gel is weak and composed of reversible, non-covalent junctions instead of permanent crosslinks, as shown by the fact that G′ is greater than G″ in most of the measured frequency window. In this system, PVA chains adhere to the bentonite basal and edge sites, forming polymer bridges between platelets that are next to each other. This creates a spatially continuous network that suppresses long-wavelength relaxation modes. This is why elasticity primarily affects the response at intermediate to high frequencies and why the terminal flow behavior is not as strong as it is with neat PVA. The drop in G′ and rise in tan δ at low frequencies suggest that the polymer bridges are breaking apart under stress and that the tactoids are rearranging. This is in line with how physically crosslinked gels break down and come back together. The time–temperature superposition principles of polymer dynamics explain why both moduli increase with frequency. These rules state that shorter deformation timescales test constrained segmental motions that are close to clay surfaces and temporary entanglements. This improves elastic storage. Pure PVA (10 wt.%) has G″ > G′ over a wide range of frequencies, on the other hand. This means that it acts as a viscoelastic liquid that is mostly composed of entanglements and does not have a network that spreads out. Another sign that no gel-like structure is formed is the lack of a low-frequency G′ plateau. These patterns fit with what is already known about colloidal gelation and how polymer–clay nanocomposites flow. These models indicate that particle bridging, percolation thresholds, and reversible physical crosslinks control the process of thick solutions transitioning to weak gels, their processability, and film-forming characteristics [52,58,59,60].

### 3.10. Thixotropic Analysis of Bentonite–PVA Dispersions

**Figure 22:**
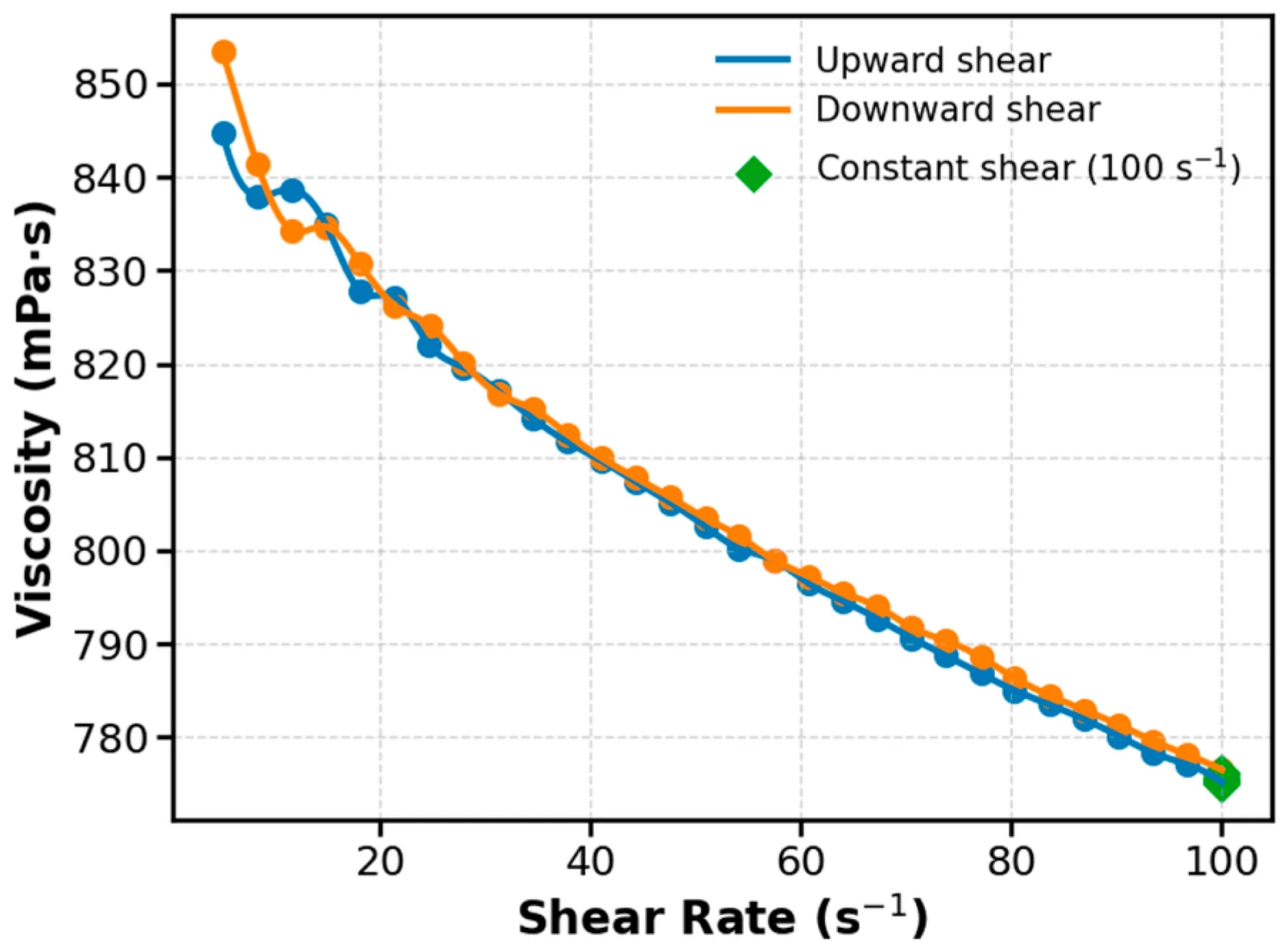
Flow curve with hysteresis loop for 10% pure PVA solution.

The viscosity of the pure PVA solution with 10% solids loading decreases smoothly and steadily as the shear rate increases (Figure 22). This occurs when polymer chains line up and then move apart. This is characteristic of shear-thinning behavior. The area under the downward shear curve was 76,487.13 mPa·s·s^−1^, and the area under the upward shear curve was 76,395.47 mPa·s·s^−1^. This yields a small hysteresis loop of 91.6 mPa·s·s^−1^. These results confirm a thixotropic response, even though the two curves are very close to each other. This happens because of interactions between the polymer network, but hydrogen bonds can break them, indicating that the damage to the structure is not irreversible. At this solids loading, the polymer matrix is so thick that it slows down the solvent’s movement. This makes the films smoother and uniform, but it also makes them take longer to dry.

**Figure 23:**
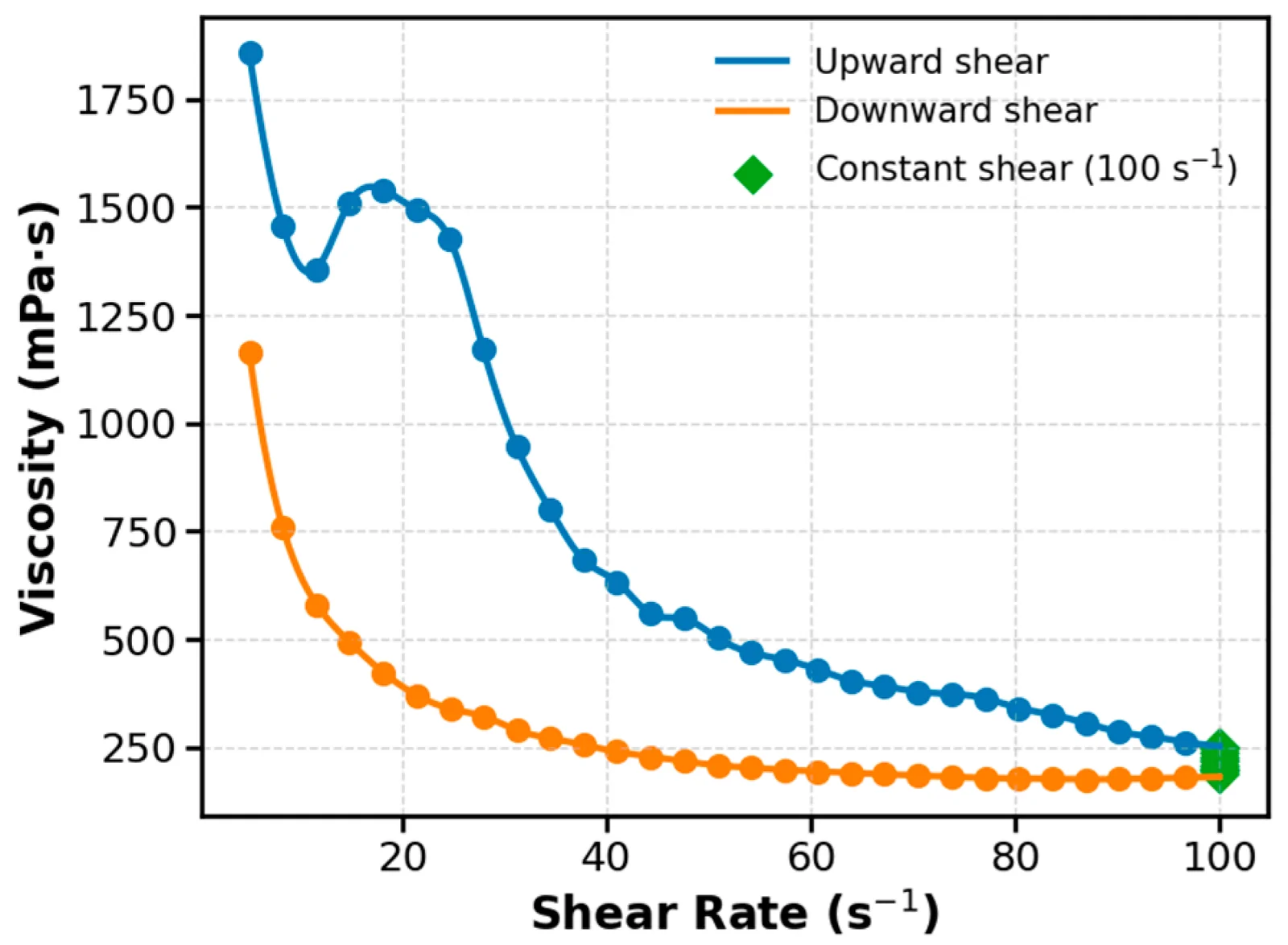
Flow curve with hysteresis loop for 5% pure bentonite solution.

The viscosity–shear rate response of the pure bentonite 5% solids aqueous suspension shows clear hysteresis behavior between the upward and downward shear ramps (Figure 23). This means that it demonstrates strong thixotropic behavior. The area under the upward shear curve was 6.79 × 10^4^ mPa·s·s^−1^, which was greater than the area under the downward shear curve (2.71 × 10^4^ mPa·s·s^−1^). The hysteresis loop area was 4.09 × 10^4^ mPa·s·s^−1^. Shear broke down the bentonite platelet network, and it did not fully recover and took a long time to do so when the shear rate was reduced. The large hysteresis curve reflects this behavior. The high viscosity at low shear rates shows that there is a strong clay network that fills the space and is held together by edge–face and electrostatic interactions. When the shear rate increases, the bentonite platelets begin to break apart and line up. This makes the viscosity drop sharply. The fact that the viscosity remains lower across the shear-rate range during the downward shear sweep shows that the clay network does not re-form right away. The hysteresis observed between the upward and downward shear curves indicates time-dependent structural changes in the 5 wt.% pure bentonite dispersion. Therefore, the observed rheological response cannot be attributed solely to shear-thinning but also reflects shear-induced structural disruption and subsequent restructuring when the applied shear conditions are changed. The extent and rate of structural recovery depend on the characteristics of the hysteresis response and the measurement conditions. Accordingly, slow structural recovery cannot be inferred solely from the presence or magnitude of thixotropic behavior.

**Figure 24:**
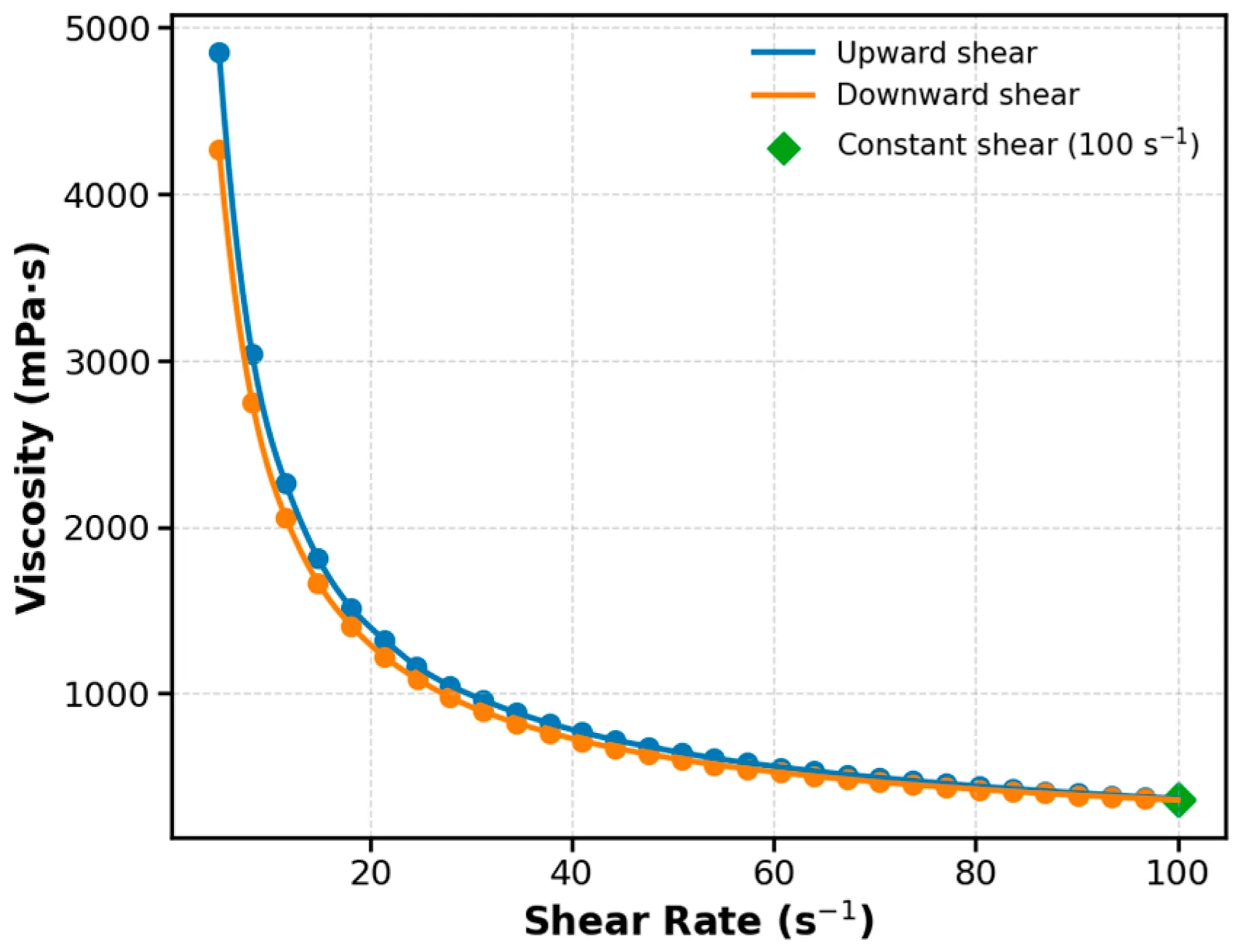
Flow curve with hysteresis loop for 10% pure bentonite solution.

The pure bentonite suspension with 10% solids loading, on the other hand, demonstrates clear hysteresis and strong thixotropic behavior (Figure 24). The area under the upward shear curve (8.81 × 10^4^ mPa·s·s^−1^) and the downward shear curve (8.16 × 10^4^ mPa·s·s^−1^) were large. The hysteresis loop was also large, at 6.54 × 10^3^ mPa·s·s^−1^. The edges and faces of the platelets are strongly connected by electrostatic forces, which leads to the formation of highly connected network of platelets. The high viscosities and big loop area provide clear evidence of this structural framework. It takes a lot of energy to slowly break up this structure, which has been percolated during the upward shear sweep. This slowly decreases the viscosity. When the shear direction is reversed, the downward curve remains below the upward curve. This means that the structure is improving over time while staying the same. The rheological response shows that bentonite suspensions with high solids contents not only exhibit shear-thinning behavior but also change over time.

**Figure 25:**
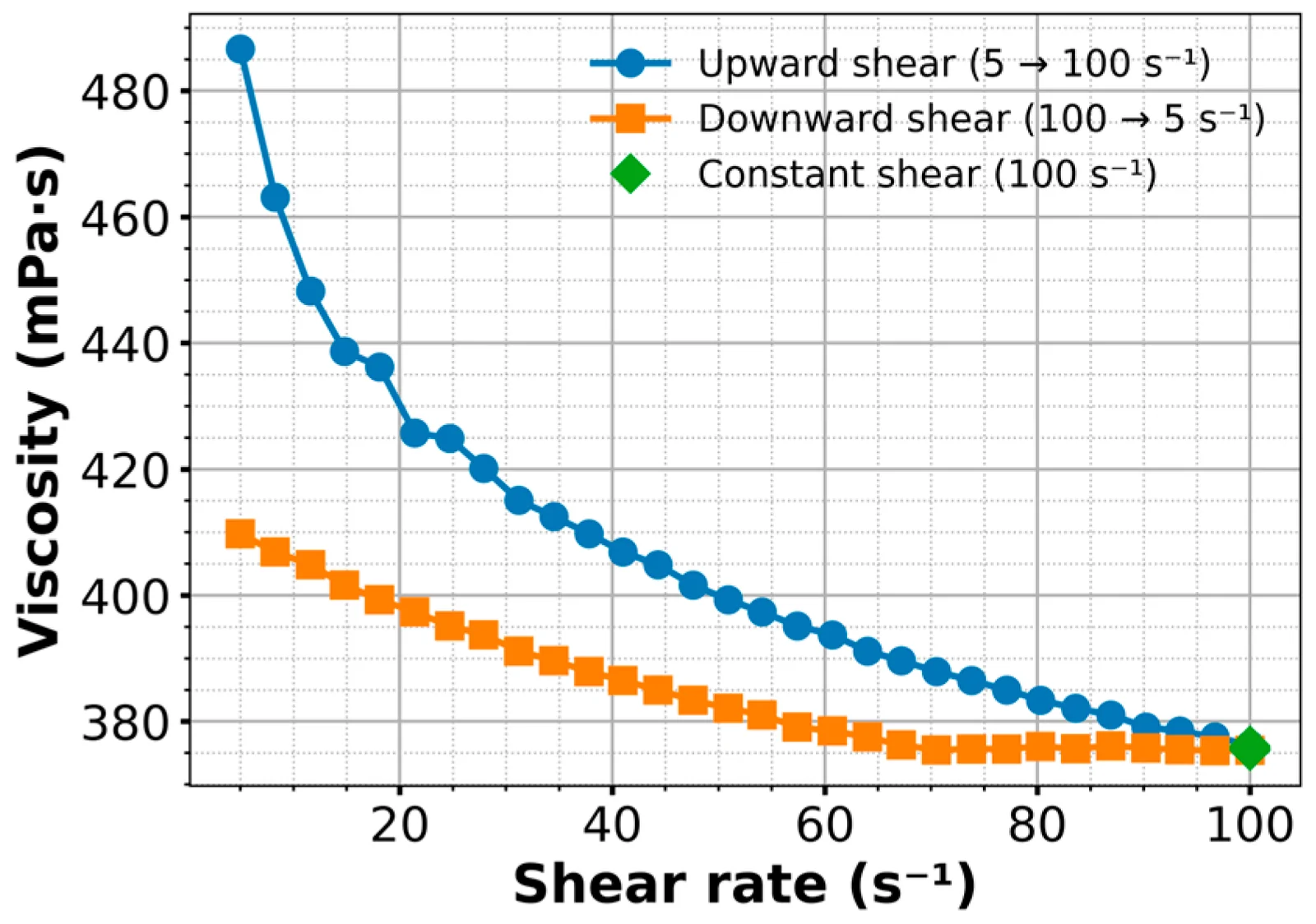
Flow curve with hysteresis loop for 25% bentonite–75% PVA with 10% solids solution.

A system with 25% bentonite and 75% PVA with 10% solids has a thixotropic response that is balanced (Figure 25). The polymer does most of the work, and the clay does not do much to help the flow. The viscosity decreases as the shear rate increases. This means that a polymer–clay network that is not very strong breaks down. For most of the shear rates, the downward shear curve stays below the upward shear curve. This means that the microstructure has not completely healed while the test is ongoing. The hysteresis loop area of about 1.43 × 10^3^ mPa·s·s^−1^ shows that the material is thixotropic. This formulation is much cheaper than what it would cost to buy pure bentonite with the same amount of solids. The highest viscosities for both sweep directions are very close to the constant-shear measurements at 100 s^−1^. This means that the microstructure is stable when sheared, and the polymer-coated platelets are arranged in a way that directs water movement. This rheological balance is advantageous for coatings because it has a low viscosity when it is applied while maintaining enough structural recovery to prevent drooping.

**Figure 26:**
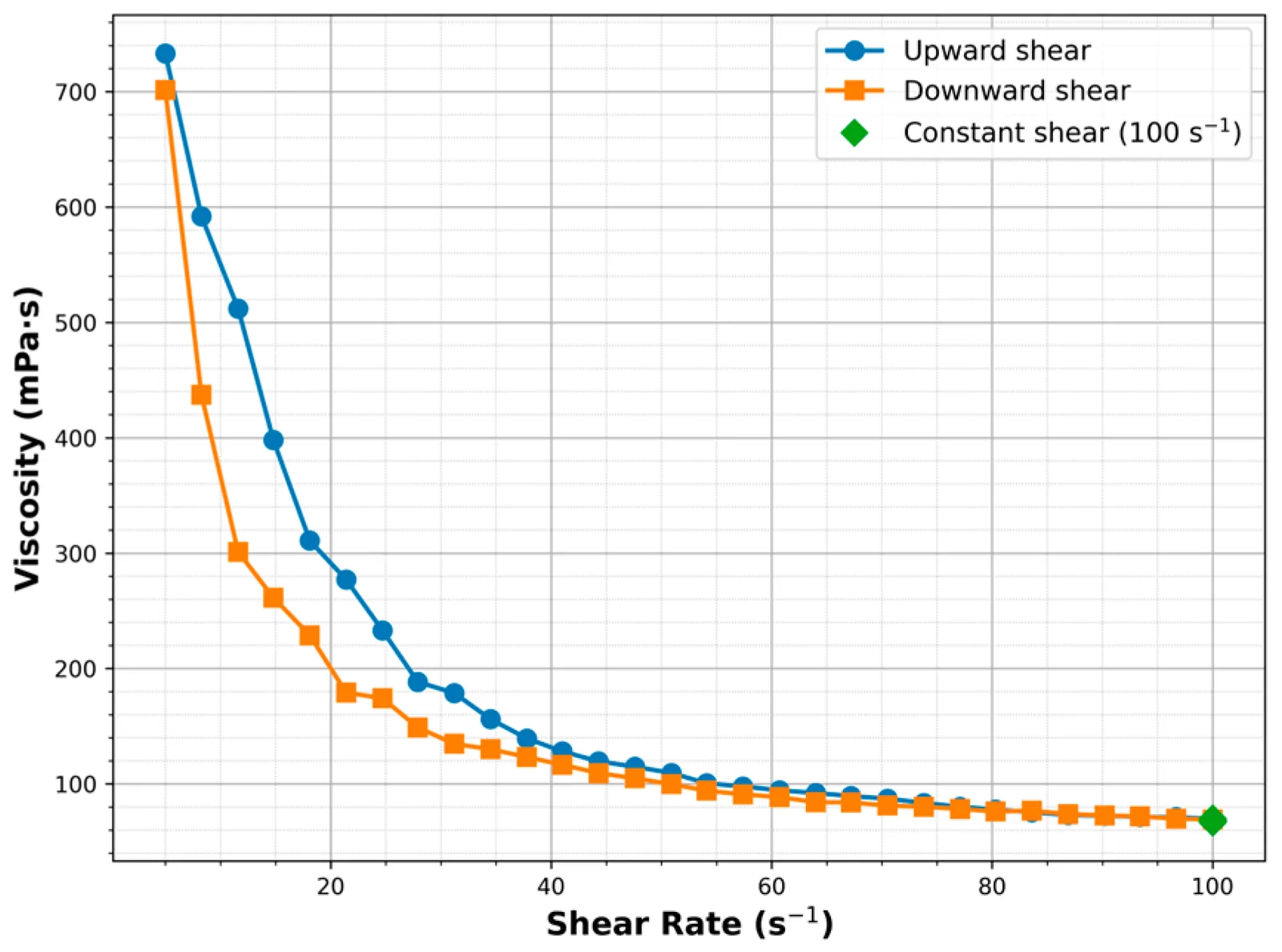
Flow curve with hysteresis loop for 50% bentonite–50% PVA with 5% solids solution.

The viscosity-shear rate profile of the 50% PVA–50% bentonite suspension with 5% solids shows a clear hysteresis loop (Figure 26). This is a sign of thixotropy, i.e., when the polymer–clay network breaks down and then builds back up over time. During the upward shear sweep, the viscosity drops quickly from 730 mPa·s at 5 s^−1^ to 70 mPa·s at 100 s^−1^. This means that the bentonite domains that were weakly flocculated and linked by hydrophilic PVA chains broke up quickly. Upon shear reversal, the downward curve remains below the upward curve. This means that the structure is still not completely healed. The hysteresis loop area, which was about 3.2 × 10^3^ mPa·s·s^−1^, shows that the material is slightly thixotropic. This behavior is characteristic of systems composed only of bentonite. The thixotropic intensity is lower because the PVA prevents the clay particles from self-aggregating and restores the network. When the shear rate is higher than about 70 s^−1^, the curves begin to converge. This means that a stable microstructure forms when shear is applied and it has minimal memory of its structure.

**Figure 27:**
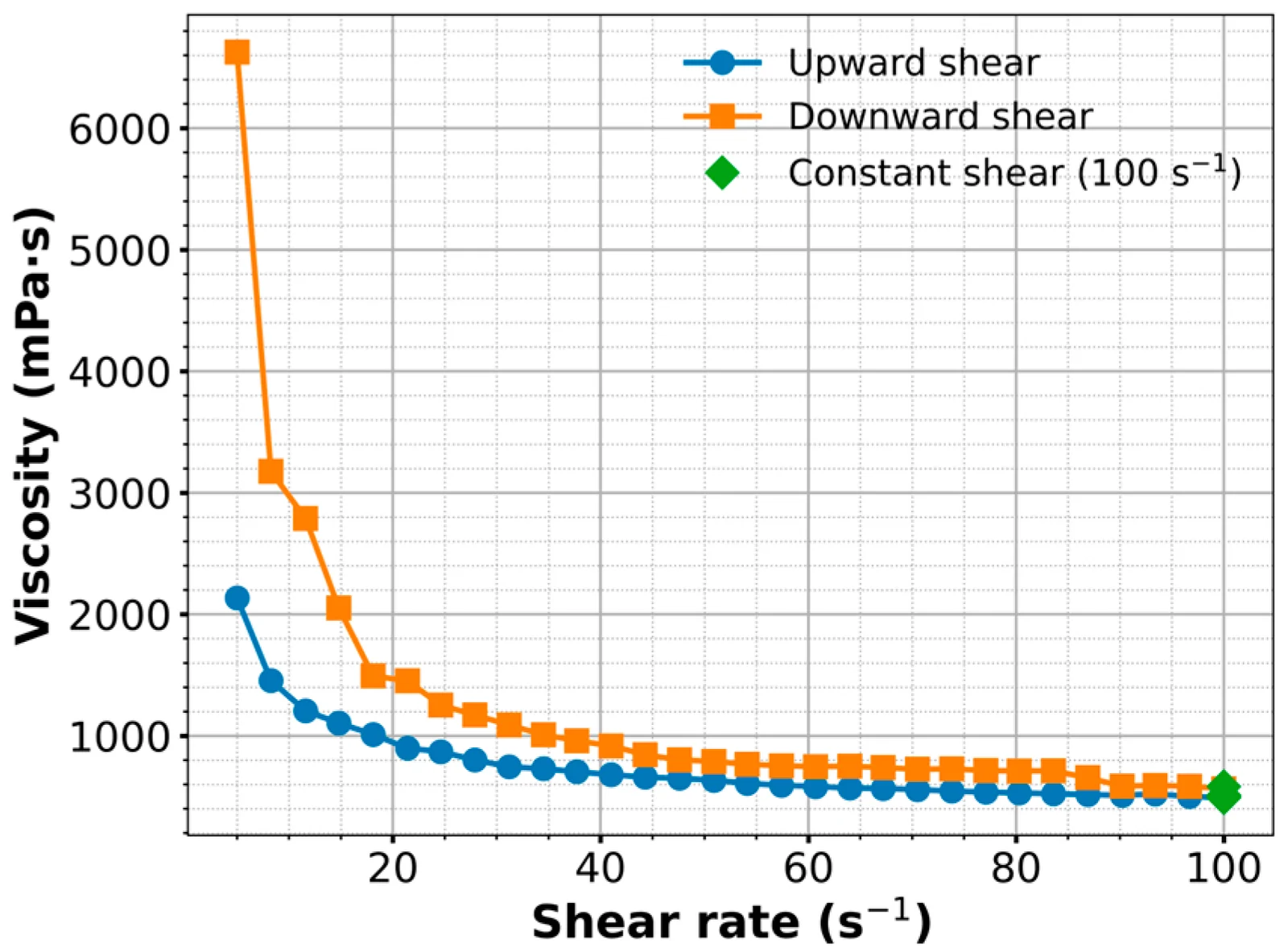
Flow curve with hysteresis loop for 50% bentonite–50% PVA with 10% solids solution.

With a solids loading of 10%, the 50% bentonite–50% PVA system has strong thixotropic properties and a hysteresis loop area of about 3.9 × 10^4^ mPa·s·s^−1^ (Figure 27). During the upward sweep, the viscosity decreases quickly from about 2135 mPa·s at low shear to about 490 mPa·s at 100 s^−1^. This means that the thick layer of clay and polymer is breaking down quickly. For most of the shear-rate range, the downward shear curve stays above the upward shear curve. This means that it takes longer for the structure to return to normal, which is very important. The viscosity stays between 485 and 500 mPa·s at 100 s^−1^. This means that a stable microstructure has formed that can handle shear. This formulation is suitable for coating applications that require fluidity while they are being applied and stay strong after they are applied. It has a stronger rheological response than systems with a high polymer content but not as strong as pure bentonite.

**Figure 28:**
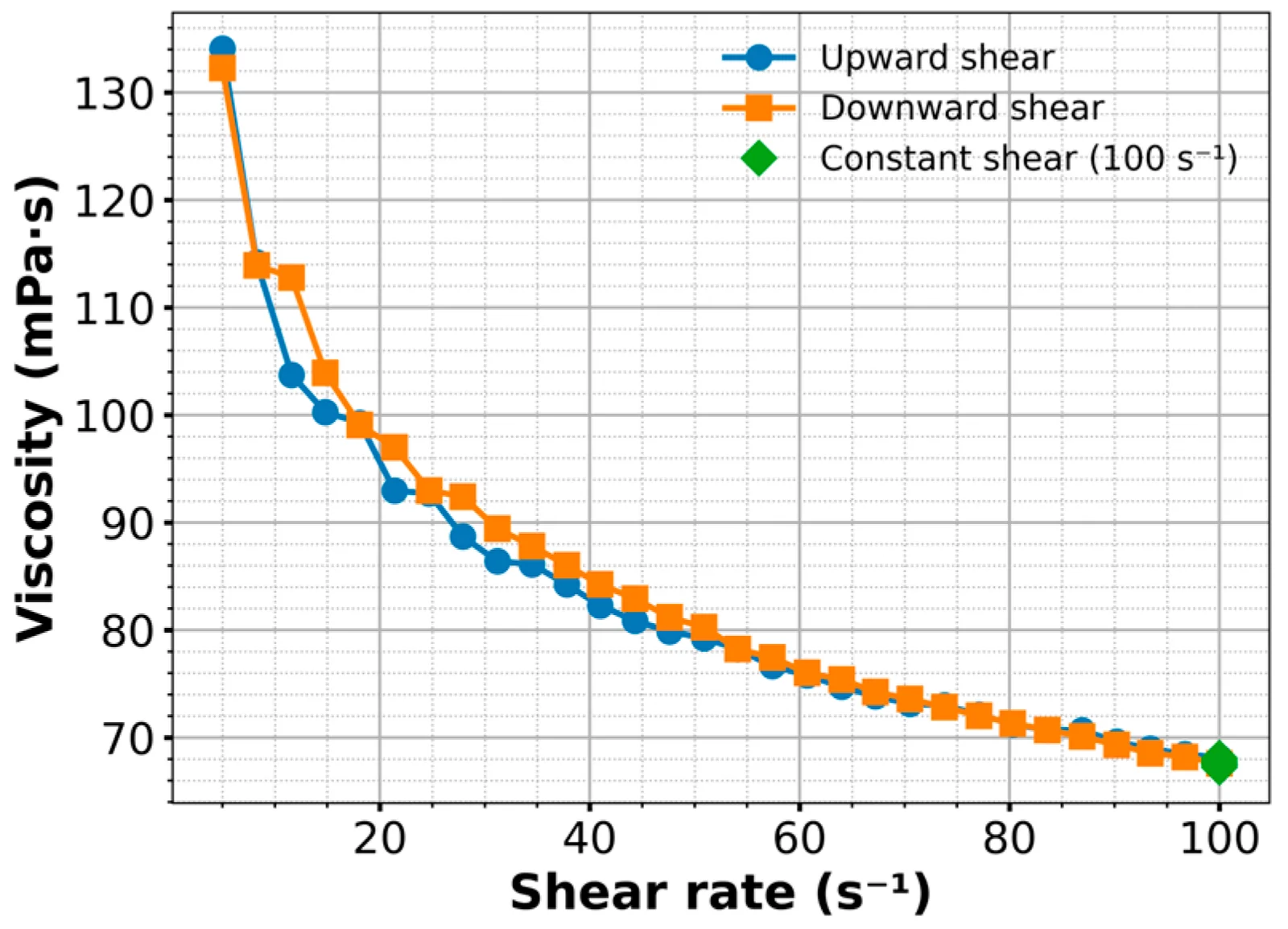
Flow curve with hysteresis loop for 75% bentonite–25% PVA with 10% solids solution.

Lastly, the system with 75% bentonite–25% PVA and 10% solids displays shear-thinning behavior without strong thixotropy (Figure 28). During the upward sweep, the viscosity decreases from about 132 mPa·s at 5 s^−1^ to about 68 mPa·s at 100 s^−1^. The constant-shear viscosity stays between 67 and 68 mPa·s at 100 s^−1^. This means that the microstructure stays stable even after being broken down by shear. The curves are so close together that they almost touch. This creates a hysteresis loop area of only about 10^2^ mPa·s·s^−1^. This weak thixotropic response means that the structure quickly settles down and does not remember what it was like before. The PVA adheres to the bentonite platelets, which prevents clay–clay interactions that cannot be reversed and speeds up the reorganization of the microstructures. This rheological profile stabilizes the shape and makes the coating easier to use. However, it might not be as resistant to sagging as formulations with higher polymer content because it has a lower recovery.

## 4. Conclusions

This study demonstrates the importance of PVA–bentonite composition and total solids content in determining the drying behavior, solvent transport, and structural evolution of polymer–clay coatings. Across the formulations investigated, drying generally occurred through two stages: an initial relatively rapid stage dominated by surface solvent evaporation, followed by a falling-rate stage associated with increasingly restricted solvent transport through the consolidating coating matrix. The relative duration and contribution of these stages varied with the PVA to bentonite ratio and solids content.

At 10 wt.% total solids, pure PVA exhibited relatively slow but comparatively uniform drying, whereas pure bentonite showed the longest drying time, reaching equilibrium after approximately 1083 min. Among the binary formulations, the 50% PVA–50% bentonite coating exhibited the shortest drying time, reaching equilibrium in approximately 480 min, together with comparatively favorable film uniformity and limited surface defects. Its thickness decreased from approximately 1745 to 440 µm during drying, corresponding to a reduction of approximately 1305 µm. By comparison, the 75%PVA–25% bentonite formulation exhibited prolonged drying and greater residual solvent, whereas the 25:75 formulation showed comparatively slower solvent removal and less uniform film formation at 5 wt.% total solids, drying was generally faster, but the resulting coatings exhibited greater microcracking and less uniform morphology. By contrast, the 10 wt.% coatings showed greater consolidation and comparatively improved film uniformity, accompanied by reduced solvent mobility.

The XRD results further indicated that both the PVA–bentonite composition and the total solids content influenced the detectable structural organization of the composite coatings. Changes in the relative intensity and breadth of the characteristic PVA and bentonite reflections were consistent with composition-dependent changes in polymer and clay organization. These observations may be associated with polymer–clay interactions and changes in clay stacking or polymer-chain ordering; however, the present XRD results do not directly confirm polymer intercalation, exfoliation, or suppression of tactoid restacking. The altered diffraction features of the 50:50 formulation, together with its drying and morphological characteristics, suggest a favorable balance between the polymer and clay phases under the investigated conditions.

Overall, the results establish a clear relationship between composition, solids content, and drying behavior in PVA–bentonite coatings. Among the formulations investigated, the 50% PVA–50% bentonite composition provided the most favorable balance of drying behavior, film formation, and rheological characteristics under the applied laboratory conditions. Replacing some of the PVA with bentonite also reduced the amount of polymer required for the formulation; however, the actual economic benefit and energy savings associated with this replacement were not quantified in the present study. A detailed techno-economic assessment and measurements of mechanical strength, adhesion, barrier performance, water resistance, and long-term durability are therefore required to establish the broader practical applicability of the formulation. These findings provide a basis for further investigation of PVA–bentonite composition and solids loading as approaches for controlling drying and film evolution in polymer–clay coating systems.

## Figures and Tables

**Table 1:** Elemental compositions of pure bentonite and pure PVA.

Element	PVA Weight (%)	PVA Atomic (%)	Bentonite Weight (%)	Bentonite Atomic (%)
C K	58.2	65.4	–	–
O K	40.2	33.9	52.47	65.77
Na K	0	0	3.03	2.64
Mg K	0	0	1.37	1.13
Al K	0	0	7.47	5.55
Si K	0.3	0.2	33.8	24.13
S K	1.1	0.5	–	–
K K	0	0	–	–
Ca K	0	0	0.79	0.39
Ti K	0.1	0	–	–
Fe L/K	0	0	1.07	0.38
Total	99.9	100	100	99.99

**Table 2:** Summary of drying data for coatings of various PVA–bentonite mixtures with 5% solids.

S. No.	Coating (Solid Constituents)	Drying Stage	Time (min)	PVA (g/cm^3^)	Bentonite (g/cm^3^)	% Solvent Left	Thickness (µm)
1	Pure PVA	Start	0	0.0506	0	100	4193
		End of Constant Rate	800	0.18	0	10	1800
		End of Falling Rate	600	0.12	0	25	2500
		Final	978	0.2382	0	17.72	1130
2	Pure Bentonite	Start	0	0	0.0499	100	4180
		End of Constant Rate	600	0	0.1403	25	2000
		End of Falling Rate	450	0	0.1001	50	2800
		Final	1094	0	0.9959	0.0059	210
3	75% PVA–25% Bentonite	Start	0	0.0376	0.0125	100	3042
		End of Constant Rate	881	0.3402	0.1131	11.0526	481
		End of Falling Rate	667	0.1994	0.0663	18.8518	706
		Final	881	0.34019	0.1131	11.0526	481
4	50% PVA–50% Bentonite	Start	0	0.0489	0.0489	100	1745
		End of Constant Rate	480	0.3335	0.3335	14.6607	440
		End of Falling Rate	364	0.2094	0.2094	23.3506	573
		Final	480	0.3335	0.3335	14.6607	440
5	25% PVA–75% Bentonite	Start	0	0.0125	0.0376	100	1800
		End of Constant Rate	600	0.0415	0.1248	30.1194	717
		End of Falling Rate	454	0.0310	0.0933	40.2890	874
		Final	600	0.0415	0.1248	30.1194	717

**Table 3:** Summary of drying data for 10% solid coatings of various PVA–bentonite mixtures.

S. No.	Coating Initial Solid Composition	Stage	Time (min)	PVA (g/cm^3^)	Bentonite (g/cm^3^)	% Solvent Left	Thickness (µm)
1	Pure PVA	Start	0	0.0385	0	100	4560
		End of Constant Rate	270	0.295	0	85	3200
		End of Falling Rate	720	0.425	0	35	1200
		Final	880	0.561	0	5	760
2	Pure Bentonite	Start	0	0	0.0421	100	4671
		End of Constant Rate	200	0	0.415	91	3000
		End of Falling Rate	700	0	0.685	67	1000
		Final	1083	0	0.835	0.7	737
3	75% PVA–25% Bentonite	Start	0	0.0376	0.0125	100	3042
		End of Constant Rate	270	0.199	0.066	18.85	706
		End of Falling Rate	667	0.34	0.113	11.05	481
		Final	881	0.34	0.113	11.05	481
4	50% PVA–50% Bentonite	Start	0	0.0489	0.0489	100	1745
		End of Constant Rate	363	0.209	0.209	23.35	573
		End of Falling Rate	480	0.333	0.333	14.66	440
		Final	480	0.333	0.333	14.66	440
5	25% PVA–75% Bentonite	Start	0	0.0125	0.0376	100	1800
		End of Constant Rate	454	0.031	0.093	40.28	874
		End of Falling Rate	600	0.0415	0.125	30.12	717
		Final	600	0.0415	0.125	30.12	717

## Data Availability

The original contributions presented in this study are included in the article/Supplementary Materials. Further inquiries can be directed to the corresponding author.

## Supplementary Materials

The following supporting information can be downloaded at: https://www.mdpi.com/article/10.3390/polym18162025/s1.
