# Supplementary material for: Mechanistic Insights into Drying and Film Evolution of PVA–Bentonite Coatings: The Role of Solids Content and Coating Composition Optimization
Source: Polymers (Basel). 2026 Aug 21;18(16):2025. doi: 10.3390/polym18162025 (PMC13517807; doi:10.3390/polym18162025)
Supplement: Supplementary file 1 [file polymers-18-02025-s001.zip › polymers-4456711-supplementary.pdf]

Supplementary Materials

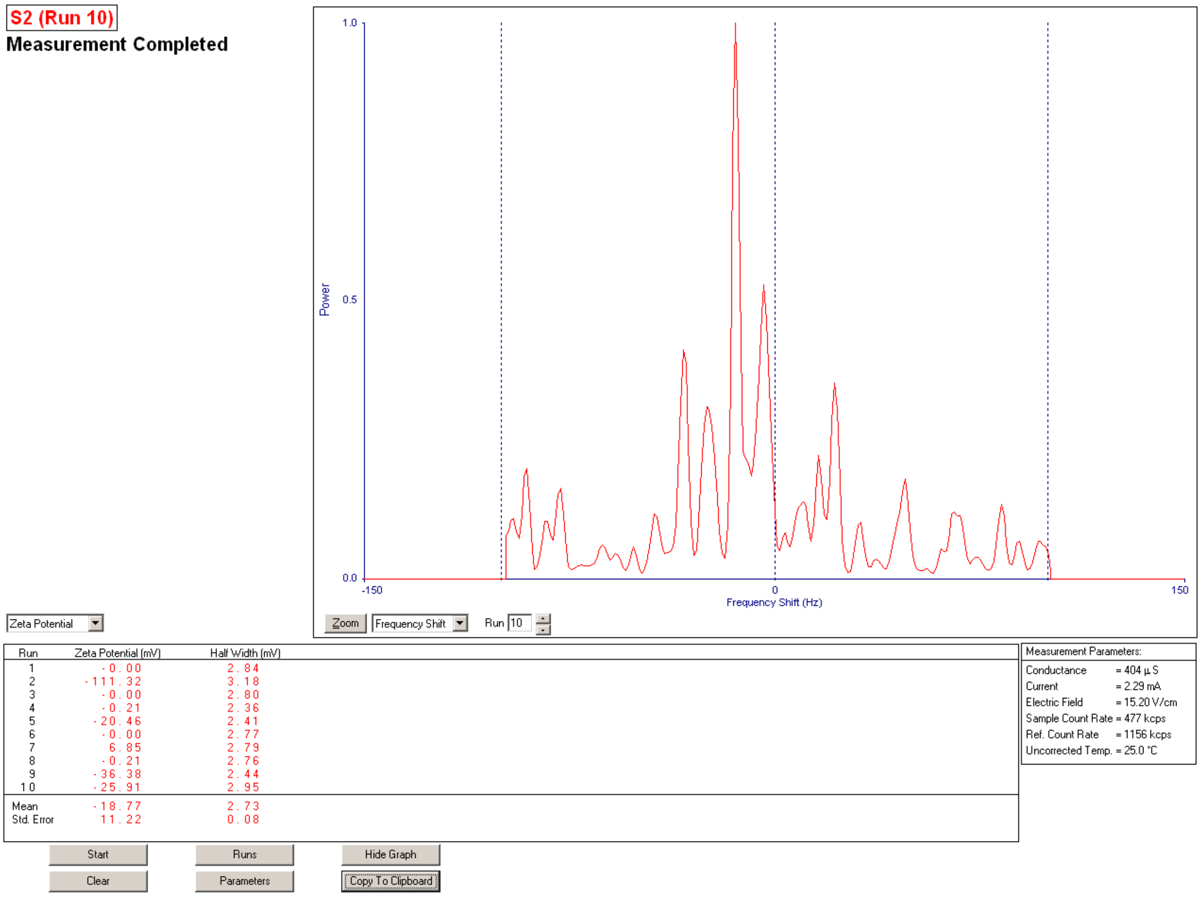

Figure S1. Zeta potential measurement of pure bentonite suspension.

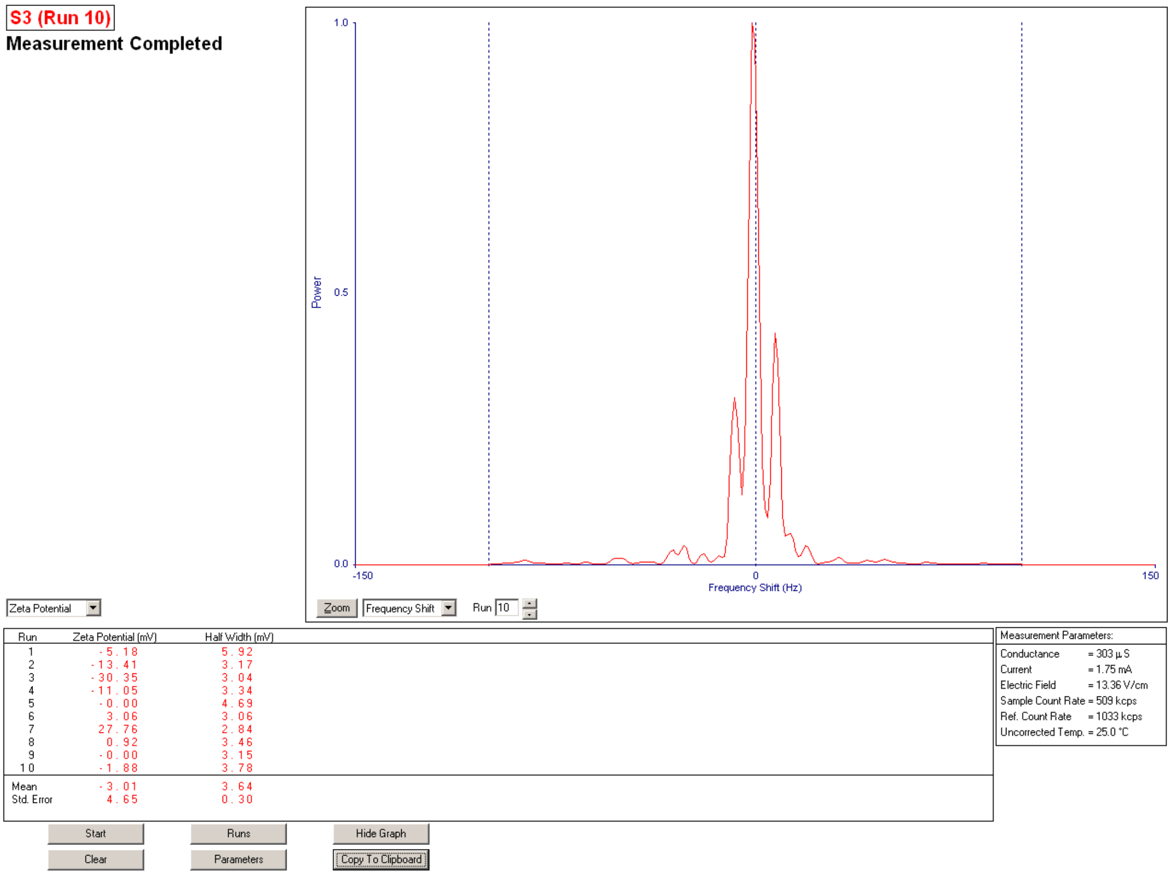

Figure S2. Zeta potential measurement of 75% bentonite – 25% PVA suspension.

S4 (Run 10)

Measurement Completed

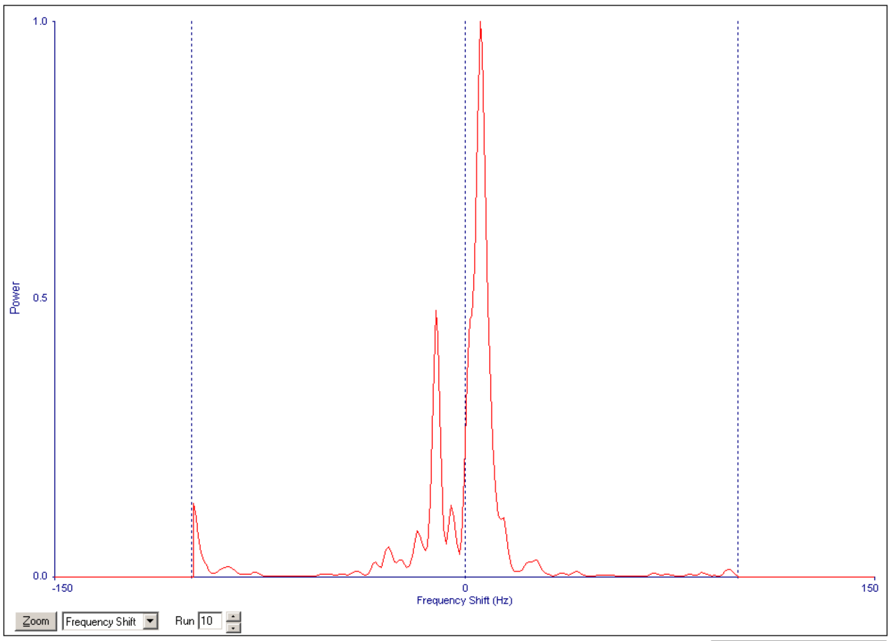

Zeta Potential

Run	Zeta Potential (mV)	Half Width (mV)
1	-16.03	2.78
2	-0.00	3.32
3	-11.39	3.51
4	3.80	3.69
5	-0.00	3.18
6	-0.96	3.57
7	-0.23	2.71
8	-21.66	4.81
9	-0.23	4.99
10	9.34	4.48
Mean	-3.74	3.70
Std. Error	3.02	0.25

Measurement Parameters:	
Conductance	= 509 µS
Current	= 2.86 mA
Electric Field	= 14.25 V/cm
Sample Count Rate	= 511 kcps
Ref. Count Rate	= 1051 kcps
Uncorrected Temp.	= 23.5 °C

Start | Runs | Hide Graph

Clear | Parameters | Copy To Clipboard

Figure S3. Zeta potential measurement of 25% bentonite – 75% PVA suspension.

S5 (Run 10)

Measurement Completed

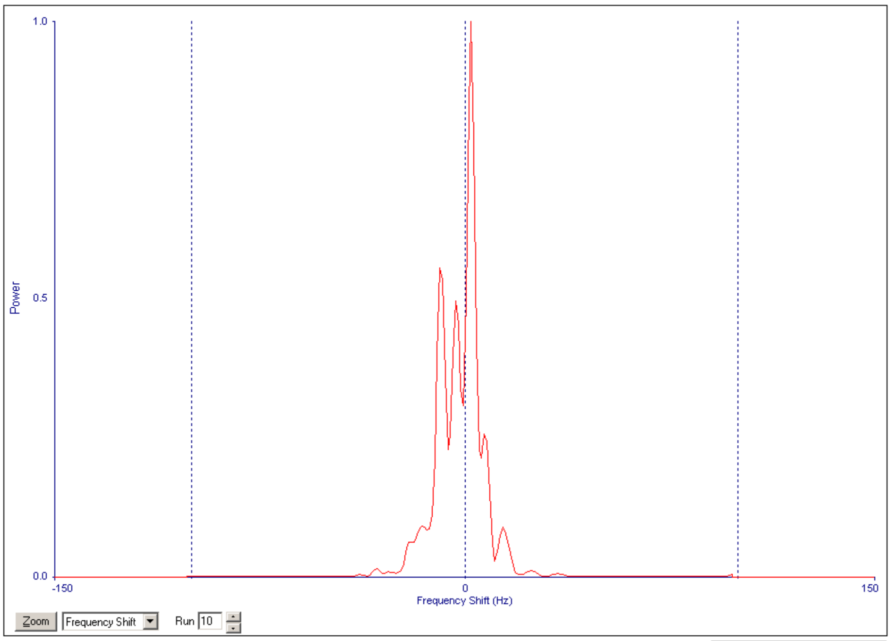

Zeta Potential

Run	Zeta Potential (mV)	Half Width (mV)
1	-159.33	3.06
2	-0.21	2.31
3	21.86	4.59
4	30.03	3.48
5	7.38	3.69
6	-0.22	2.48
7	13.62	7.19
8	0.90	3.23
9	-4.69	3.73
10	5.08	3.85
Mean	-8.56	3.76
Std. Error	17.10	0.44

Measurement Parameters:

Conductance = 988 µS
Current = 5.45 mA
Electric Field = 15.03 V/cm
Sample Count Rate = 384 kcps
Ref. Count Rate = 929 kcps
Uncorrected Temp. = 23.5 °C

Start | Runs | Hide Graph
Clear | Parameters | Copy To Clipboard

Figure S4. Zeta potential measurement of 50% bentonite – 50% PVA suspension.
